# Acoustically induced transparency for synchrotron hard x-ray photons

**DOI:** 10.1038/s41598-021-86555-x

**Published:** 2021-04-12

**Authors:** I. R. Khairulin, Y. V. Radeonychev, V. A. Antonov, Olga Kocharovskaya

**Affiliations:** 1 Institute of Applied Physics, Russian Academy of Sciences, Nizhny Novgorod, 603950 Russia; 2 N. I. Lobachevsky State University of Nizhny Novgorod, Nizhny Novgorod, 603950 Russia; 3 Kazan Physical-Technical Institute, Russian Academy of Sciences, Kazan, 420029 Russia; 4 Department of Physics and Astronomy, Institute for Quantum Studies and Engineering, Texas A&M University, College Station, TX 77843-4242 USA

**Keywords:** X-rays, Single photons and quantum effects

## Abstract

The induced transparency of opaque medium for resonant electromagnetic radiation is a powerful tool for manipulating the field-matter interaction. Various techniques to make different physical systems transparent for radiation from microwaves to x-rays were implemented. Most of them are based on the modification of the quantum-optical properties of the medium under the action of an external coherent electromagnetic field. Recently, an observation of acoustically induced transparency (AIT) of the ^57^Fe absorber for resonant 14.4-keV photons from the radioactive ^57^Co source was reported. About 150-fold suppression of the resonant absorption of photons due to collective acoustic oscillations of the nuclei was demonstrated. In this paper, we extend the AIT phenomenon to a novel phase-locked regime, when the transmitted photons are synchronized with the absorber vibration. We show that the advantages of synchrotron Mössbauer sources such as the deterministic periodic emission of radiation and controlled spectral-temporal characteristics of the emitted photons along with high-intensity photon flux in a tightly focused beam, make it possible to efficiently implement this regime, paving the way for the development of the acoustically controlled interface between hard x-ray photons and nuclear ensembles.

## Introduction

Some appealing advantages of the hard x-ray photons with energies of 10–100 keV resonantly interacting with atomic nuclei, compared to optical photons resonantly interacting with bound electrons in atoms, stimulate the development of quantum x-ray optics^1–3^. For example, 14.4-keV radiation can be focused into a several nanometers spot and e-times attenuated by just 70 nm-thick ^57^Fe foil at room temperature^4,5^. Recently, a number of fundamental quantum-optics effects were implemented in this frequency range (see^1–3,6–19^ and references therein). However, most methods that are well suited for controlling the light-matter interaction in the infrared and visible ranges are currently ineffective or unfeasible for hard x-rays. This is mainly due to the absence of sufficiently intense spectrally narrow coherent sources and high-finesse cavities in the hard x-ray/γ-ray range^19^.

At the same time, other opportunities emerge. One of them, providing the basis for the Mössbauer nuclear spectroscopy, is to use the Doppler effect by means of mechanical movement of an absorbing medium relative to a source of radiation. This opportunity is due to the short wavelength of radiation and narrow Mössbauer (recoilless) spectral lines of quantum transitions in nuclei. For example, motion of ^57^Fe absorber at a constant velocity of 0.17 mm/s along the photon propagation direction shifts the position of its 1.13 MHz-wide spectral line, centered at wavelength of 0.86 Å, by 2 MHz. Another well-known manifestation of the Doppler effect is the periodic modulation of the quantum transition frequencies of nuclei in the case of acoustic vibration of the absorber. If the vibration frequency exceeds the transition linewidth, such a modulation leads to splitting of a recoilless absorption single line into a comb of equidistant well-resolved spectral components separated by the vibration frequency (see^19–24^ and references therein). The basic consequence of this multi-frequency nuclear response is the appearance of sidebands in the spectrum of the transmitted field, accompanied by a decrease in the absorption of radiation at the nuclear resonance and an increase in the integral transmittance of the absorber^19,25–28^. If the absorber vibrates as a whole (piston-like vibration), the produced sidebands can be synchronized, which leads to their periodic constructive interference and transformation of quasi-monochromatic γ-radiation into a regular sequence of short pulses. One can control the spectral and temporal characteristics of pulses via variation of the frequency, amplitude, and initial phase of vibration, as well as constant velocity of the source relative to the absorber^6,29–31^. It is worth noting that a similar modulation of atomic transition frequencies in the VUV/XUV range can be induced by a strong infrared or optical field via the Stark effect, leading to the transformation of quasi-monochromatic XUV or VUV radiation into extremely short pulses^32–34^.

Recently, a different effect of the piston-like vibration of the ^57^Fe recoilless absorber on the resonant propagation of 14.4-keV recoilless photons from the ^57^Co radioactive Mössbauer source (RMS) was demonstrated^19^. It was shown that collective oscillations of nuclei with certain values of amplitudes and frequencies can greatly reduce the resonant interaction of quasi-monochromatic radiation with an optically deep two-level nuclear absorber in such a way, that the transmitted photons preserve their energy, spectral profile and the temporal waveform (the time dependence of the photon detection probability, proportional to the photon count rate or the intensity of the single-photon wave packet)^19^. This effect of the acoustically induced transparency (AIT) for quasi-monochromatic γ-ray photons^35,36^ is an analog of electromagnetically induced transparency (EIT) and Autler-Townes splitting (ATS) in optics, which are the widely used tools for realization of quantum interfaces between single optical photons and atomic ensembles^19,37–42^.

In the proof-of-principle experiment^19^, AIT was implemented with 14.4-keV photons, which were stochastically emitted by RMS in a random phase of the absorber vibration. The present work aims at studying a new regime of AIT, when the emission of the single-photon wave packet and the vibration of the absorber are synchronized. This phase-locked regime is crucial for realization of an acoustically controlled interface between individual x-ray photons and nuclear ensembles, since it leads to interference effects and introduces a relative vibration phase as an important control parameter. For example, in the phase-locked regime, an acoustic switch from the transparency for an incident photon to its transformation into a series of the short pulses^6,29^ may be implemented in the same experimental set up by changing the absorber vibration amplitude and the frequency detuning between the absorber and the source. The acoustically induced photon delay line via resonant dispersion accompanying AIT^19^ could be another example of an acoustically controlled interface.

Periodic modulation of the nuclear transition frequency by uniform nuclear vibrations results in a drastic modification of the quantum state of the nuclear ensemble, replacing each energy state with a set of quasi-energy states (Floquet state) the positions, populations, and relative phases of which are controlled by the frequency, amplitude, and phase of the vibrations^43^. As a result, instead of exponential absorption accompanied by dynamic beats in an optically thick absorber^44,45^, the transmitted photon experiences a dramatically different change in its state. Its waveform may be qualitatively modified, or transmitted without change, or shifted in time as a whole^6,19,29–31^. A number of new opportunities, including possible realization of nuclear EIT and the EIT-based nuclear quantum memory, can emerge in the multilevel nuclear system with hyperfine splitting of the ground and excited energy levels, when the hyperfine splitting is a multiple of the vibration frequency, so that the Floquet states corresponding to the different original eigenstates of the system overlap and interfere.

In this paper, we show that interference effects in the phase-locked regime of AIT lead to a regular modulation of the photon waveform, which depends on the initial phase of the absorber vibration. We determine the optimal value of the initial vibration phase minimizing this waveform modulation, and show that the maximum amplitude of this modulation decreases with increasing modulation frequency. We also show that in the case of poor AIT, realized at a relatively low frequency of the absorber vibration, the modulation of the photon waveform can be reduced by using dual-tone vibration.

It should be noted that the implementation of the phase-locked regime of AIT with RMS is challenging and inefficient. This is due to the fact that each stochastically emitted photon corresponds to a random phase of the absorber vibration. Therefore, locking of the photon emission to a certain phase of the absorber vibration can only be approximately simulated by means of cutting off all emitted photons, except for those that correspond to a small phase interval in the vicinity of the given vibration phase. This leads to a dramatic decrease in the resulting photon flux, accompanied by an increase in the background (which degrades the data quality) and an increase in the accumulation time.

The phase-locked regime of AIT can be naturally implemented using a ^57^Fe Synchrotron Mössbauer Source (SMS) available at ESRF and Spring-8 facilities^46–53^. The SMS constitutes a near-perfect ^57^FeBO_3_ (iron borate) single crystal that extracts quasi-monochromatic radiation from broadband x-ray bursts emitted from the storage ring of a synchrotron facility. It is implemented via pure nuclear Bragg scattering of synchrotron radiation (SR) into an energy band of 14.4-keV quantum transitions of ^57^Fe nucleus with a little background. All photons produced by the SMS can be synchronized with vibration of the absorber due to periodic nature and deterministic timing of the SR pulses. Synchronization implies that the absorber vibration frequency is a multiple of the SR pulse repetition rate, which, in turn, is a multiple of the bunch revolution frequency in the synchrotron storage ring. In contrast to RMS producing photons with the Lorentz spectral line and 98 ns duration of the single-photon wave packet (measured as a full width at half maximum, FWHM), the SMS can produce about three times shorter Lorentz-line photons as well as photons with variable spectral-temporal profiles and durations^46–53^. As an example, we consider SMS-photon with Lorentz-squared spectral profile^47–50^. It corresponds to the gradually growing pulse front, which is required for realization of nuclear quantum memories^16^. We show that at the same conditions, the higher quality AIT can be realized for SMS-photons than for RMS-photons. We also show that the phase dependence of the photon waveform distortions is less pronounced for the Lorentz-squared photon and the distortion value in the Lorentz-squared photon waveform is as small as for the Lorentzian photon in the case of the optimal vibration phase.

It should be noted that acoustic control requires a uniform amplitude of nuclear oscillations across a photon beam, which can be implemented by reducing the beam cross-section. Thus, a micrometer-sized beam of SMS-photons along with high photon flux and a suitable spectral-temporal structure of radiation, constitute the crucial advantages of SMS for the implementation of acoustic control and, specifically, the phase-locked regime of AIT.

The paper is organized as follows. Theoretical model describes the theoretical model. In AIT in the phase-locked regime for Lorentzian photon and AIT in the phase-locked regime for Lorentz-squared photon, we consider the AIT of the ^57^Fe absorber in the phase-locked regime for x-ray single-photon wave packet emitted by SMS with Lorentz and Lorentz-squared spectral lines. For both line shapes, we discuss the optimal conditions to minimize alterations in the spectral and temporal characteristics of photons transmitted through the vibrating resonant absorber. In AIT in the phase-locked regime via dual-tone vibration, we study the possibility of improving the spectral-temporal characteristics of the transmitted photons by using dual-tone vibration of the absorber. In Conclusion, we summarize the results.

## Theoretical model

**Figure 1:**
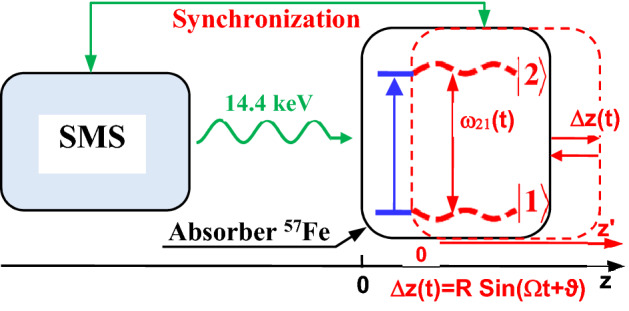
(Color online) Energy scheme of the resonant interaction between 14.4-keV photons and vibrating absorber. Recoilless 14.4-keV photons (λ≈0.86 Å) emitted from SMS (left side) resonantly interact with transition |1> →|2> of ^57^Fe nuclei during the propagation through the single-line ^57^Fe absorber (right side). They are resonantly absorbed in motionless absorber (blue lines). Harmonic vibration of the absorber as a whole (piston-like vibration) with circular frequency Ω, amplitude *R*, and initial phase *ϑ* along the photon propagation direction (marked in red) or dual-tone vibration leads to periodic temporal variation in |1> →|2> transition frequency *ω*_21_ (*t*) (dashed red curves) due to the Doppler effect. The emission of photons can be locked to the initial phase of the absorber vibration, *ϑ.* The axis *z* labels the laboratory reference frame, red axis *z*' labels the reference frame of the vibrating absorber, and Δ*z* = *z–z*'.

The AIT, similar to other effects of photon propagation in the vibrating resonant absorber, can be described on the basis of the same model and the same set of equations as in^6,16–31,44,45^. We discuss the following experimental scheme (Fig. 1).

The 14.4 keV photons are emitted by SMS or RMS and propagate in the *z*-direction to a ^57^Fe absorber located at *z* = 0. An ensemble of photons from a source can be described as a quasi-monochromatic single-photon wave packet with the electric field in the following form (see^6,19–31,44,45^ and references therein),

1 $$E_{S} (t,z \leqslant 0) = \overline{E}_{S} \left( {t - {\raise0.5ex\hbox{$\scriptstyle z$} \kern-0.1em/\kern-0.15em \lower0.25ex\hbox{$\scriptstyle c$}}} \right)e^{{ - i\omega_{SC} \left( {t - {\raise0.5ex\hbox{$\scriptstyle z$} \kern-0.1em/\kern-0.15em \lower0.25ex\hbox{$\scriptstyle c$}}} \right) + i\varphi }},$$

where $$\omega_{SC}$$ and $$\overline{E}_{S} \left( {t - {\raise0.5ex\hbox{$\scriptstyle z$} \kern-0.1em/\kern-0.15em \lower0.25ex\hbox{$\scriptstyle c$}}} \right)$$ is the carrier frequency and the envelope of the wave packet, $$\varphi$$ is the random initial phase of the field, and $$c$$ is the speed of light in vacuum. At the entrance to the absorber, $$z = 0$$, the boundary condition is

2 $$E_{S} (t,z = 0) = \overline{E}_{S} (t)e^{{ - i\omega_{SC} t + i\varphi }},$$

where no reflection of the incident field from the absorber surface is assumed. The envelope of the wave packet, $\overline{E}_{S} (t)$, corresponds to the profile of the source radiation spectral line, $\tilde{E}_{S} (\delta_{S} )$, according to the Fourier transform,

3 $$\tilde{E}_{S} (\delta_{S} )\, = \frac{1}{2\pi }\int\limits_{ - \infty }^{\infty } {dt\;\overline{E}_{S} (t)e^{{ - i\delta_{S} t + i\varphi }} \,} ,$$

where $\delta_{S} = \omega_{SC} - \omega_{S}$ is the detuning of the Fourier constituent at frequency $\omega_{S}$ from the carrier frequency. The two-level absorber with quantum transition frequency $\omega_{21}$ sinusoidally vibrates as a whole (piston-like vibration) along *z*-axis,

4 $$z = z^{\prime} + R\sin (\Omega t + \vartheta ) ,$$

where $z^{\prime}$ is the coordinate in the vibrating reference frame (Fig. 1), Ω, *R*, and *ϑ* are the circular frequency, amplitude, and initial phase of vibration, respectively. Equation (4) implies that the physical thickness of the absorber, *L*, is much less than the wavelength of sound, $L < < {{2\pi V_{sound} } \mathord{\left/ {\vphantom {{2\pi V_{sound} } \Omega }} \right. \kern-\nulldelimiterspace} \Omega }$ (where $V_{sound}$ is the speed of sound in the absorber) and its motion is non-relativistic, $R\Omega < < c$.

As follows from Eqs. (1) and (4), *ϑ* indicates the absorber vibration phase at the moment of incidence of the single-photon wave packet. Owing to the periodic emission of the SR pulses, the initial vibration phase, *ϑ,* can be locked at an arbitrary selected value via tuning a feedback-loop circuit that synchronizes the absorber vibration with a timing signal from the storage ring. This restricts the choice of the vibration frequency, Ω, by the discrete values multiple of the SR pulse repetition rate^54^. The SR pulse repetition rate depends on the operation mode of the facility^46,51^ and is a multiple of the synchrotron revolution frequency. The revolution frequency is 355.04 kHz at the ESRF^46^ and 208.81 kHz at the Spring-8 facility^51^.

**Figure 2:**
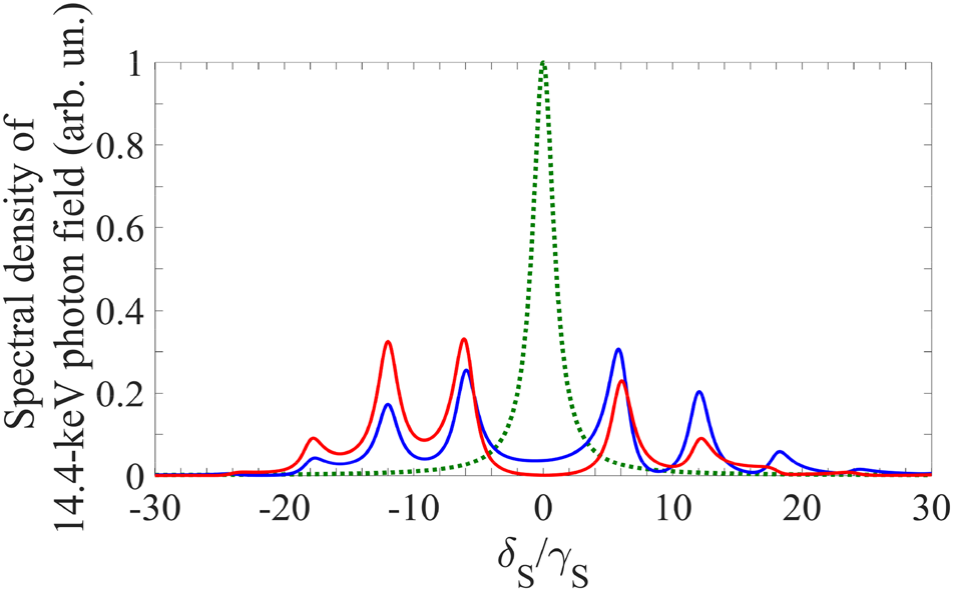
(Color online) Spectrum of an incident photon and the absorption spectral profile of the nuclear transition in the case of harmonically vibrating absorber under the AIT condition (Eq. 7). Green dotted curve is the spectral profile of the incident field in the case of Lorentz source's spectral line with halfwidth* γ*_*S*_ [the normalized modulus squared of Eq. (18)] in the laboratory reference frame. In the reference frame of the vibrating absorber, the green dotted curve turns into a blue (at $\vartheta = 0$) or red (at $\vartheta = \pi /2$) curve, which are plotted according to Eqs. (6) and (18) as $\left| {\tilde{E^{\prime }_{S}} (\delta_{S} ,0)} \right|^{2}$ at $\Omega /\gamma_{S} = 6$, $p = p_{1} = 2.4$, and $\omega_{SC} = \omega_{21}$. In the case of Lorentz-squared source spectral line (AIT in the phase-locked regime for Lorentz-squared photon) the spectrum at arbitrary $\vartheta$ in the vibrating reference frame is qualitatively similar to the red curve. The green dotted curve also represents the Lorentz spectral line of the absorber transition |1> →|2> in the vibrating reference frame, if its halfwidth, $\gamma_{21}$, equals $\gamma_{S}$ [plotted as the normalized modulus squared of Eq. (9b) at $\omega_{SC} = \omega_{21}$].

The resonant interaction of recoilless x-ray or γ-ray radiation with vibrating nuclear absorber can be considered either in the laboratory frame of reference or in the reference frame co-moving with the absorber. The laboratory reference frame is more convenient for studying the response of the absorber to the electromagnetic field, while the reference frame of the vibrating absorber is most suitable for analyzing the spectral-temporal transformations of the field^6,19–31^. According to (Eq. 4), in the vibrating reference frame, the field (Eqs. 1, 2) of a single-photon wave packet is seen by the nuclei as a frequency-modulated field,

5 $$E^{\prime}_{S} (t,z^{\prime} \leqslant 0) = \overline{E}_{S} \left( {t{{ - z^{\prime}} \mathord{\left/ {\vphantom {{ - z^{\prime}} c}} \right. \kern-\nulldelimiterspace} c}} \right)e^{{ - i\omega_{SC} (t{{ - z^{\prime}} \mathord{\left/ {\vphantom {{ - z^{\prime}} c}} \right. \kern-\nulldelimiterspace} c}) + i\varphi + ip\sin \left( {\Omega t + \vartheta } \right)}},$$

where $p = 2\pi {R \mathord{\left/ {\vphantom {R {\lambda_{SC} }}} \right. \kern-\nulldelimiterspace} {\lambda_{SC} }}$ is the index of modulation of the carrier frequency and $\lambda_{SC} = {{2\pi c} \mathord{\left/ {\vphantom {{2\pi c} {\omega_{SC} }}} \right. \kern-\nulldelimiterspace} {\omega_{SC} }}$ is the corresponding wavelength. In Eq. (5), we neglected modulation in the envelope of the photon field due to the non-relativistic motion of the absorber. According to (Eq. 3), using the Jacobi-Anger expansion, $e^{ \pm ip\sin \phi } = \sum\limits_{n = - \infty }^{\infty } {J_{n} (p)e^{ \pm in\phi } }$ (where $J_{n} (p)$, $n \in {\mathbb{Z}}$ is the *n*-th Bessel function of the first kind), one can represent the spectrum of the field (Eq. 5) in the vibrating reference frame as a superposition of the spectral lines of incident radiation (Eq. 3), shifted from the carrier frequency by a frequency multiple of the vibration frequency^6,19–31^,

6 $$\tilde{E^{\prime}_{S}} (\delta_{S} ,0) = \sum\limits_{n = - \infty }^{\infty } {\tilde{E}_{S} (\delta_{S} + n\Omega )\,J_{ - n} (p)e^{ - in\vartheta } } = \sum\limits_{n = - \infty }^{\infty } {\tilde{E}_{S}^{(n)} (\delta_{S} + n\Omega )} ,$$

where $\delta_{S} + n\Omega = \omega_{SC} + n\Omega - \omega_{S}$ is the detuning of the Fourier constituent at frequency $\omega_{S}$ of the *n*-th spectral line from its central frequency $\omega_{SC} + n\Omega$. As can be seen from (Eq. 6), if the vibration frequency exceeds the halfwidth of the source line, $\Omega > \gamma_{S}$ (where $\left| {\tilde{E}_{S} (\omega_{SC} \pm \gamma_{S} )} \right|^{2} = {{\left| {\tilde{E}_{S} (\omega_{SC} )} \right|^{2} } \mathord{\left/ {\vphantom {{\left| {\tilde{E}_{S} (\omega_{SC} )} \right|^{2} } 2}} \right. \kern-\nulldelimiterspace} 2}$), the spectral line of the source looks like a phase-matched spectral comb in the reference frame of the vibrating absorber (Fig. 2). Both the spectral amplitude and spectral phase of the Fourier constituent with frequency $\omega_{S}$ of the total field, $\tilde{E^{\prime}_{S}}$, are determined by the amplitude and initial phase of the absorber vibration via all coefficients $J_{ - n} (p)e^{ - in\vartheta }$ in the superposition.

Let us consider the case when the frequency of the absorber quantum transition, $\omega_{21}$, coincides with the carrier frequency of the incident field, $\omega_{21} = \omega_{SC}$, while the vibration amplitude, $R$, takes the value

7 $$R = R_{i} ,{\text{where}}\quad R_{1} \approx 0.38\lambda_{SC} ,R_{2} \approx 0.88\lambda_{SC} ,R_{3} \approx 1.37\lambda_{SC} ,...$$

This is the first condition for AIT^19^, at which the parent (zero) spectral line vanishes for all frequencies, $\tilde{E}_{S}^{(0)} \approx 0$, due to $J_{0} (p_{i} = 2\pi R_{i} /\lambda_{SC} ) \approx 0$, $i = 1,2,3...$ in (Eq. 6). As a result, in the vibrating reference frame, the most part of the photon energy is spread among the sidebands, $\tilde{E}_{S}^{(n)}$, located out of resonance, (Fig. 2). However, the wings of sidebands remain in resonance. For example, the residual field $\tilde{E^{\prime}_{S}} (0,0) \equiv \tilde{E^{\prime}_{S}} (\delta_{S} = 0,z^{\prime} = 0)$ in (Eq. 6) at frequency $\omega_{S} = \omega_{SC} = \omega_{21}$ (Fig. 2) is

8 $$\tilde{E^{\prime}_{S}} (0,0) = \sum\limits_{n = - \infty ,n \ne 0}^{\infty } {\tilde{E}_{S} (n\Omega )\,J_{ - n} (p_{i} )e^{ - in\vartheta } } .$$

At frequencies near nuclear resonance, the wings of the sidebands are absorbed and acquire a phase incursion during the field propagation. This leads to incomplete transparency, accompanied by a decrease in the transmitted energy and distortions in the outgoing single-photon wave packet. The energy loss, as well as the shape and value of distortions are determined by the total in-resonance field, which, according to (Eq. 8), is the result of interference of all sidebands at resonance, which depends on the initial phase of vibration, $\vartheta$.

The transformation of each Fourier constituent of the incident field, $\tilde{E^{\prime}_{S}} (\delta_{S} ,0)$ in (Eq. 6), at frequency $\omega_{S}$, into $\tilde{E^{\prime}_{S}} (\delta_{S} ,L)$ at the absorber exit, $z^{\prime} = L$ is well known (see^6,19–44^ and references therein). It corresponds to the Beer–Lambert–Bouguer law. In the case of a homogeneously broadened |1> →|2> absorber transition with a halfwidth of its spectral line $\gamma_{21}$, the Fourier constituent with frequency $\omega_{S}$ of the outgoing field can be written as the incident field multiplied by the corresponding exponential term,

9a $$\tilde{E^{\prime}_{S}} (\delta_{S} ,L) = e^{{{{ - [T_{e} + T_{M} \eta_{21} (\delta_{S} )]} \mathord{\left/ {\vphantom {{ - [T_{e} + T_{M} \eta_{21} (\delta_{S} )]} 2}} \right. \kern-\nulldelimiterspace} 2}}} \tilde{E^{\prime}_{S}} (\delta_{S} ,0) ,$$

9b $$\eta_{21} (\delta_{S} ) = \frac{1}{{1 + {{i\left( {\omega_{21} - \omega_{SC} + \delta_{S} } \right)} \mathord{\left/ {\vphantom {{i\left( {\omega_{21} - \omega_{SC} + \delta_{S} } \right)} {\gamma_{21} }}} \right. \kern-\nulldelimiterspace} {\gamma_{21} }}}},$$

where $T_{e}$ is the exponent factor of photoelectric absorption (which is omitted in evaluations below for simplicity) and $T_{M} = f_{LM} \sigma NL$ is the Mössbauer thickness of the absorber resonant transition corresponding to the optical depth of a medium ($f_{LM}$ is the Lamb-Mössbauer factor accounting for the probability of recoilless absorption, $\sigma$ is the nuclear resonant cross section at |1> →|2> transition, and $N$ is the concentration of ^57^Fe nuclei in the absorber). The inverse Fourier transform of (Eq. 9a) determines the temporal form of the single-photon wave packet behind the absorber in the vibrating reference frame,

10 $${E^{\prime}_S}(t,z^{\prime} \geqslant L) = {e^{{{ - {T_e}} \mathord{\left/ {\vphantom {{ - {T_e}} 2}} \right. \kern-\nulldelimiterspace} 2}}}{e^{ - i{\omega _{SC}}\left( {t - {{z^{\prime}} \mathord{\left/ {\vphantom {{z{\prime}} c}} \right. \kern-\nulldelimiterspace} c}} \right)}}\int\limits_{ - \infty }^\infty {d{\delta _S}\;{e^{i{\delta _S}\left( {t - {{z{\prime}} \mathord{\left/ {\vphantom {{z{\prime}} c}} \right. \kern-\nulldelimiterspace} c}} \right)}}\,{e^{{{ - {T_M}{\eta _{21}}({\delta _S})} \mathord{\left/ {\vphantom {{ - {T_M}{\eta _{21}}({\delta _S})} 2}} \right. \kern-\nulldelimiterspace} 2}}}\sum\limits_{n = - \infty }^\infty {{{\tilde E}_S}({\delta _S} + n\Omega )\,{J_{ - n}}(p){e^{ - in\vartheta }}} } .$$

Using the relation (Eq. 4) between *z*′ and $z$ in the above approximations gives a field in the laboratory reference frame,

11 $$E_{S} (t,z \geqslant L) = e^{{{{ - T_{e} } \mathord{\left/ {\vphantom {{ - T_{e} } 2}} \right. \kern-\nulldelimiterspace} 2}}} e^{{ - i\omega_{SC} \left( {t - {z \mathord{\left/ {\vphantom {z c}} \right. \kern-\nulldelimiterspace} c}} \right)}} e^{{ - ip\sin \left( {\Omega t + \vartheta } \right)}} \int\limits_{ - \infty }^{\infty } {d\delta_{S} \;e^{{i\delta_{S} \left( {t - {z \mathord{\left/ {\vphantom {z c}} \right. \kern-\nulldelimiterspace} c}} \right)}} \,e^{{{{ - T_{M} \eta_{21} (\delta_{S} )} \mathord{\left/ {\vphantom {{ - T_{M} \eta_{21} (\delta_{S} )} 2}} \right. \kern-\nulldelimiterspace} 2}}} \sum\limits_{n = - \infty }^{\infty } {\tilde{E}_{S} (\delta_{S} + n\Omega )\,J_{ - n} (p)e^{ - in\vartheta } } }.$$

As can be seen from Eqs. (10) and (11), the intensity, $I_{out} (t) = \frac{c}{8\pi }\left| {E^{\prime}_{S} (t,z^{\prime} = L)} \right|^{2}$, of the outgoing single-photon field in the vibrating reference frame is also the intensity of the single-photon wave packet in the laboratory reference frame^6,19,30,31^,

12 $$I_{out} (t) = \frac{c}{8\pi }e^{{ - T_{e} }} \left| {\int\limits_{ - \infty }^{\infty } {d\delta_{S} \;e^{{i\delta_{S} t}} \,e^{{{{ - T_{M} \eta_{21} (\delta_{S} )} \mathord{\left/ {\vphantom {{ - T_{M} \eta_{21} (\delta_{S} )} 2}} \right. \kern-\nulldelimiterspace} 2}}} \sum\limits_{n = - \infty }^{\infty } {\tilde{E}_{S} (\delta_{S} + n\Omega )\,J_{ - n} (p)e^{ - in\vartheta } } } } \right|^{2} .$$

The intensity (Eq. 12) determines the measured waveform of the transmitted single photon, i.e., the time dependence of the photon detection probability proportional to the photon count rate. The spectrum of the transmitted single-photon wave packet in the laboratory reference frame can be obtained by the Fourier transform of the field (Eq. 11) (see also Supplemental Material in^19^).

13 $$\eqalign{ & {{\tilde E}_S}({\delta _S},z = L) = {e^{{{ - {T_e}} \mathord{\left/ {\vphantom {{ - {T_e}} 2}} \right. \kern-\nulldelimiterspace} 2}}}{e^{i{\omega _{SC}}L/c}}\sum\limits_{q,n = - \infty }^\infty {{J_{n + q}}(p){J_q}(p){e^{ - in\vartheta }}{{\tilde E}_S}({\delta _S} + n\Omega )\,\exp \left\{ { - {{{T_M}{\eta _{21}}\left[ {{\delta _S} + \left( {n + q} \right)\Omega } \right]} \mathord{\left/ {\vphantom {{{T_M}{\eta _{21}}\left[ {{\delta _S} + \left( {n + q} \right)\Omega } \right]} 2}} \right. \kern-\nulldelimiterspace} 2}} \right\}.} \cr}$$

Equations (9a)–(13) describe the spectral and temporal transformations of the single-photon wave packet of an arbitrary envelop in the vibrating resonant absorber. As follows from Eqs. (9a)–(13), the transformations are determined by the detuning between the carrier frequency of the photon and the resonant frequency of the absorber, as well as by the frequency, amplitude, and initial phase of the absorber vibration, and the absorber optical depth. These equations were used to study transformation of the exponentially decreasing waveform of 14.4-keV γ-ray photon with stepwise front edge, emitted by the ^57^Co radioactive source, into a regular sequence of ultrashort pulses^6,29–31^ as well as to describe some properties of AIT for such photons in ^57^Fe vibrating absorber^19^.

The transformation from Eqs. (5) to (10) of an arbitrarily shaped single-photon wave packet in a vibrating absorber can be described directly in the time domain via the response function technique^48,55–58^. In the absorber reference frame, the transmitted field at the output of the absorber, $E^{\prime}_{out} (t) \equiv E^{\prime}_{S} (t,z^{\prime} = L)$, can be calculated as the convolution integral of the incident field (Eq. 5), $E^{\prime}_{in} (t) \equiv E^{\prime}_{S} (t,z^{\prime} = 0)$, and the absorber response function $R(t)$,

14 $$E^{\prime}_{out} (t) = \int\limits_{ - \infty }^{\infty } {R\left( {t - \tau } \right)E^{\prime}_{in} \left( \tau \right)d\tau },$$

with (see, for example^45^)

15 $$R\left( q \right) = e^{{ - T_{e} /2}} \left[ {\delta \left( q \right) - \frac{{T_{M} \gamma_{21} }}{2}\theta \left( q \right)e^{{ - \left( {i\omega_{21} + \gamma_{21} } \right)q}} {{J_{1} \left( {2\sqrt {\frac{{T_{M} \gamma_{21} }}{2}q} } \right)} \mathord{\left/ {\vphantom {{J_{1} \left( {2\sqrt {\frac{{T_{M} \gamma_{21} }}{2}q} } \right)} {\sqrt {\frac{{T_{M} \gamma_{21} }}{2}q} }}} \right. \kern-\nulldelimiterspace} {\sqrt {\frac{{T_{M} \gamma_{21} }}{2}q} }}} \right] ,$$

where $\delta (q)$ is the Dirac delta function. Then the intensity (Eq. 12) of the transmitted field can be calculated also as

16 $$I_{out} (t) = \frac{c}{8\pi }\left| {\int\limits_{ - \infty }^{\infty } {R\left( {t - \tau } \right)E^{\prime}_{in} \left( \tau \right)d\tau } } \right|^{2}.$$

## AIT in the phase-locked regime for Lorentzian photon

The SMS can produce 14.4-keV x-ray photons with various spectral and temporal characteristics depending on the temperature of the ^57^FeBO_3_ crystal, its angular position relative to the incident SR beam near the Bragg angle, as well as on the applied external constant and radiofrequency magnetic fields^47–53^. In particular, at room temperature the SMS can produce a spectrum of six polarized Lorentz lines corresponding to the Zeeman-split 14.4-keV ^57^Fe transition. In this case a single line similar to the Lorentz line of ^57^Co RMS can be extracted^47,48^. For both these sources, the corresponding single-photon wave packet (Eq. 1) has an exponentially decreasing envelop with a stepwise front edge,

17 $$\overline{E}_{S} \left( {t - {z \mathord{\left/ {\vphantom {z c}} \right. \kern-\nulldelimiterspace} c}} \right) = E_{0} \theta \left( {t - {z \mathord{\left/ {\vphantom {z c}} \right. \kern-\nulldelimiterspace} c}} \right)e^{{ - \gamma_{S} \left( {t - {z \mathord{\left/ {\vphantom {z c}} \right. \kern-\nulldelimiterspace} c}} \right)}},$$

where $\theta \left( x \right)$ is the Heaviside step function, and $\gamma_{S}$ is the halfwidth of the SMS spectral line. As discussed in^47,48^, the measured minimum width of the SMS spectral line is $2\gamma_{S} \approx 2.9\Gamma_{0}$, where $\Gamma_{0} /(2\pi ) \approx 1.13{\text{ MHz}}$ is the natural linewidth of the 14.4-keV ^57^Fe transition. It is about 2.4 times wider than the typical linewidth of RMS, $2\gamma_{S} \approx 1.2\Gamma_{0}$^19^. Hence, one can intuitively expect that the implementation of AIT with characteristics similar to^19^ should require a higher vibration frequency. Let us estimate its value from the following spectral analysis.

According to (Eq. 3), the spectrum of the incident and transmitted field in the vibrating reference frame is described by formulae (Eq. 6) and (Eq. 9a), respectively, with the line shape

18 $$\tilde{E}_{S} (\delta_{S} ) = \frac{{\tilde{E}_{S} (0)}}{{1 + {{i\delta_{S} } \mathord{\left/ {\vphantom {{i\delta_{S} } {\gamma_{S} }}} \right. \kern-\nulldelimiterspace} {\gamma_{S} }}}} ,$$

where $\tilde{E}_{S} (0) = E_{0} e^{i\varphi } /(2\pi \gamma_{S} )$. In the case (Eq. 7) of AIT, the resonant part, $\omega_{S} = \omega_{SC} = \omega_{21}$, of spectrum (Eq. 9a) at the exit of the absorber can be represented as

19 $$\tilde{E^{\prime}_{S}} (0,L) = e^{{{{ - T_{e} } \mathord{\left/ {\vphantom {{ - T_{e} } 2}} \right. \kern-\nulldelimiterspace} 2}}} e^{{{{ - T_{M} } \mathord{\left/ {\vphantom {{ - T_{M} } 2}} \right. \kern-\nulldelimiterspace} 2}}} \tilde{E^{\prime}_{S}} (0,0) ,$$

where the resonant frequency constituent, $\tilde{E^{\prime}_{S}} (0,0)$, is described by Eq. (8) with substituting (18). It can be rewritten as

20 $$\begin{gathered} \tilde{E^{\prime}}_{S} (0,0) = 2\tilde{E}_{S} (0)\sum\limits_{n = 1}^{\infty } {\frac{{J_{2n} (p_{i} )}}{{1 + (2n\Omega /\gamma_{S} )^{2} }}\left\{ {\,\cos (2n\vartheta ) - \frac{2n\Omega }{{\gamma_{S} }}\sin (2n\vartheta )} \right\}} \hfill \\ { + }i\frac{{J_{2n - 1} (p_{i} )}}{{1 + \left[ {(2n - 1)\Omega /\gamma_{S} } \right]^{2} }}\left\{ {\,\,\sin \left[ {(2n - 1)\vartheta } \right] + \frac{(2n - 1)\Omega }{{\gamma_{S} }}\cos \left[ {(2n - 1)\vartheta } \right]} \right\}. \hfill \\ \end{gathered}$$

**Figure 3:**
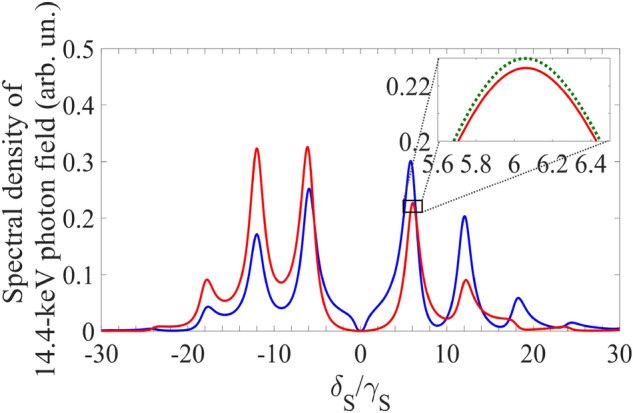
(Color online) Spectrum of the single-photon wave packet with Lorentz spectral shape, transmitted through the vibrating absorber under the AIT condition (Eq. 7) in the absorber reference frame. Blue and red curves correspond to those in Fig. 2. They are plotted as the normalized function $\left| {\tilde{E^{\prime}}_{S} (\delta_{S} ,L)} \right|^{2}$ in Eq. (9a) for $T_{e} = 0$, $T_{M} = 5$, $\gamma_{S} /\gamma_{21} = 3$, and $\tilde{E}_{S} (\delta_{S} )$ in the form of (Eq. 18) at the same as in Fig. 2 parameter values ($\omega_{SC} = \omega_{21}$, $p = p_{1}$, $\Omega /\gamma_{S} = 6$) for $\vartheta = 0$ (the blue curve) and $\vartheta = {\pi \mathord{\left/ {\vphantom {\pi 2}} \right. \kern-\nulldelimiterspace} 2}$ (the red curve). The inset shows the maximum difference between the transmitted field and the incident field (the green dotted curve, the same as the red solid curve in Fig. 2), occurring near the peak of the first sideband.

As follows from (Eq. 20), at $p = p_{1} \approx 2.4$, the total resonant part of the sideband wings, $\left| {\tilde{E^{\prime}_{S}} (0,0)} \right|$, has the maximum value at approximately $\vartheta = k\pi$ and has the minimum value at approximately $\vartheta = (2k - 1)\pi /2$, $k \in {\mathbb{Z}}$ (see also Fig. 2),

$$\tilde{E^{\prime}_{S}} (0,0)_{{{\text{max}}}}^{{}} \approx 2\tilde{E}_{S} (0)\left\{ {\frac{{J_{2} (p_{1} )}}{{1 + (2\Omega /\gamma_{S} )^{2} }} \mp i\frac{{J_{1} (p_{1} )\Omega /\gamma_{S} }}{{1 + \left( {\Omega /\gamma_{S} } \right)^{2} }}} \right\} ,$$

21 $$\tilde{E^{\prime}_{S}} (0,0)_{{{\text{min}}}} \approx - 2\tilde{E}_{S} (0)\left\{ {\frac{{J_{2} (p_{1} )}}{{1 + (2\Omega /\gamma_{S} )^{2} }} \mp i\frac{{J_{1} (p_{1} )}}{{1 + (\Omega /\gamma_{S} )^{2} }}} \right\},$$

due to constructive and destructive interference of the sideband wings at the resonance frequency, respectively. Here, minus corresponds to odd *k* and plus corresponds to even *k*. One can see from Eq. (21) that $\left| {\tilde{E^{\prime}_{S}} (0,0)} \right|_{{{\text{max}}}} = O\left( {\gamma_{S} /\Omega } \right)$ and $\left| {\tilde{E^{\prime}_{S}} (0,0)} \right|_{{{\text{min}}}} = O\left( {\gamma_{S}^{2} /\Omega^{2} } \right)$. Therefore, the proper adjusting the initial phase of the absorber vibration to the moments of SR emission can minimize the energy loss and spectral-temporal distortions of the transmitted radiation. In the case $\vartheta = {\pi \mathord{\left/ {\vphantom {\pi 2}} \right. \kern-\nulldelimiterspace} 2}$, the output field spectrum (Fig. 3) is closer to the incident field spectrum shown in Fig. 2, than in the case $\vartheta = 0$, which corresponds to better transparency.

As can be seen from Fig. 2, the Lorentz wings of the nuclear spectral line (green dotted curve) overlap with the field sidebands and therefore affect them. This influence is illustrated in the inset of Fig. 3. In the first order by ${{T_{M} \gamma_{21} } \mathord{\left/ {\vphantom {{T_{M} \gamma_{21} } {(2\Omega )}}} \right. \kern-\nulldelimiterspace} {(2\Omega )}}$, the transmitted field at the frequency of the *m*-th sideband's maximum, $m\Omega$ ($m \in {\mathbb{Z}}$, $m \ne 0$), can be estimated from Eq. (9a) as

22 $$\tilde{E^{\prime}_{S}} (m\Omega ,L) \approx e^{{{{ - T_{e} } \mathord{\left/ {\vphantom {{ - T_{e} } 2}} \right. \kern-\nulldelimiterspace} 2}}} \tilde{E}_{S} (0)\,\left\{ {1 - i\frac{{T_{M} \gamma_{21} }}{2m\Omega }} \right\}J_{ - m} (p_{i} )e^{ - im\vartheta },$$

where the first term in brackets is the incident field at frequency $m\Omega$, and the second term describes the alteration caused by the incompletely suppressed influence of the absorber resonant transition on the propagating field caused mainly by its dispersion (Fig. 3 inset). This influence does not depend on the initial phase of vibration and can be eliminated only via increasing the vibration frequency or reducing the linewidth and optical depth of the absorber. Thus, Eqs. (20) and (22) determine the two more conditions of AIT^19^ for arbitrary initial phase of the absorber vibration,

23 $${{\gamma_{S} } \mathord{\left/ {\vphantom {{\gamma_{S} } \Omega }} \right. \kern-\nulldelimiterspace} \Omega } \ll 1,{{T_{M} \gamma_{21} } \mathord{\left/ {\vphantom {{T_{M} \gamma_{21} } {(2\Omega )}}} \right. \kern-\nulldelimiterspace} {(2\Omega )}} \ll 1 .$$

At the same time, as follows from Eqs. (21) and (22), if conditions (Eq. 23) are met within the first order by ${{\gamma_{S} } \mathord{\left/ {\vphantom {{\gamma_{S} } \Omega }} \right. \kern-\nulldelimiterspace} \Omega }$, then at $\vartheta = {\pi \mathord{\left/ {\vphantom {\pi 2}} \right. \kern-\nulldelimiterspace} 2}$ the dependence of the distortions in the spectral-temporal characteristics of the transmitted field for a given value of the vibration frequency, $\Omega$, on the source linewidth is negligible. This, in particular, means that if the vibration phase of the ^57^Fe absorber relative to the front of the 14.4-keV single-photon wave packet is locked to π/2, the transparency with SMS having a triple natural linewidth will not be worse than transparency with RMS having a natural linewidth under the same other conditions.

As an example, we consider the ^57^Fe absorber with a natural linewidth $\gamma_{21} = \Gamma_{0} /2$ and optical depth $T_{M} = 5$ piston-like vibrated at frequency ${\Omega \mathord{\left/ {\vphantom {\Omega {(2\pi )}}} \right. \kern-\nulldelimiterspace} {(2\pi )}} \approx 10.2{\text{ MHz}}$ (so that $\Omega /\gamma_{21} = 18$). These parameters are similar to those used in^19^ with RMS. Besides, the chosen vibration frequency is close to some specific values that can be used in phase-locked experiments at the ESRF (for example, 9.94 MHz at the 4-bunch filling mode^46^) or at the Spring-8 facility (for example, 10.23 MHz at the 1/7-filling + 5 bunches filling mode^51^).

**Figure 4:**
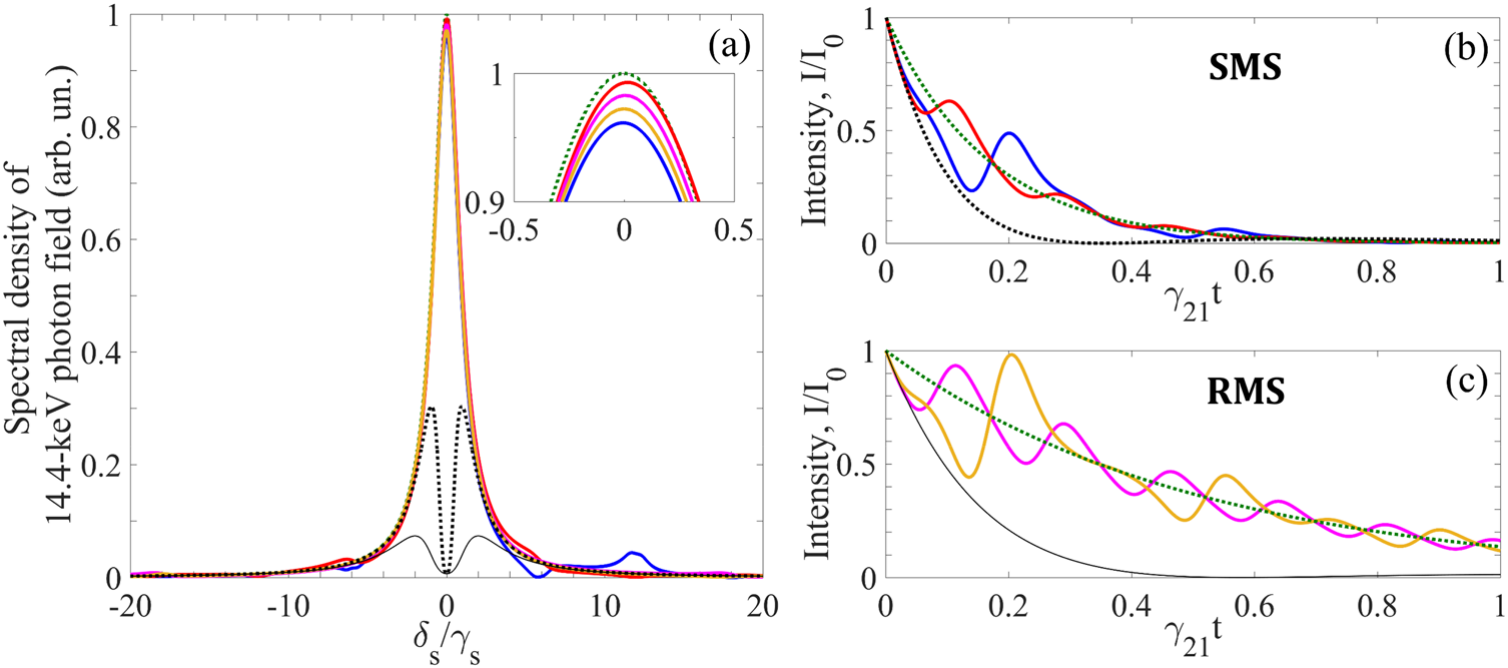
(Color online) Spectrum **(a)** and waveform **(b,c)** of the Lorentzian single-photon wave packet transmitted through the ^57^Fe absorber ($T_{M} = 5$, $T_{e} = 0$) vibrated with frequency ${\Omega \mathord{\left/ {\vphantom {\Omega {(2\pi )}}} \right. \kern-\nulldelimiterspace} {(2\pi )}} \approx 10.2{\text{ MHz}}$ corresponding to $\Omega /\gamma_{21} = 18$ (as in Fig. 3) in the laboratory reference frame for SMS ($\gamma_{S} = 3\gamma_{21}$, blue and red lines for $\vartheta = 0$ and $\vartheta = {\pi \mathord{\left/ {\vphantom {\pi 2}} \right. \kern-\nulldelimiterspace} 2}$, respectively) and for RMS ($\gamma_{S} = \gamma_{21}$, orange and purple lines for $\vartheta = 0$ and $\vartheta = {\pi \mathord{\left/ {\vphantom {\pi 2}} \right. \kern-\nulldelimiterspace} 2}$, respectively). The green dotted lines correspond to the incident photon, whereas the black dotted and solid lines—to the SMS and RMS photon, respectively, transmitted through the motionless absorber. The blue, red, and green lines correspond to those in Figs. 2 and 3. The blue, red, purple, orange and black curves are plotted as the normalized function $\left| {\tilde{E}_{S} (\omega_{S} ,z = L)} \right|^{2}$ in Eq. (13) for **(a)** and as the function $I_{out} (t)/I_{0}$ [where $I_{0} = {{cE_{0}^{2} } \mathord{\left/ {\vphantom {{cE_{0}^{2} } {(8\pi )}}} \right. \kern-\nulldelimiterspace} {(8\pi )}}$] in Eq. (12) for **(b,c)**, with $\tilde{E}_{S} (\delta_{S} )$ taken from Eq. (18) under the parameter values in Fig. 3.

In this case, if SMS has the tripled natural linewidth ($\gamma_{S} = 3\gamma_{21}$ as in Fig. 2, Fig. 3 and hereafter), the AIT conditions in (Eq. 23) are met poorly: ${{\gamma_{S} } \mathord{\left/ {\vphantom {{\gamma_{S} } \Omega }} \right. \kern-\nulldelimiterspace} \Omega } = {1 \mathord{\left/ {\vphantom {1 6}} \right. \kern-\nulldelimiterspace} 6}$, ${{T_{M} \gamma_{21} } \mathord{\left/ {\vphantom {{T_{M} \gamma_{21} } {(2\Omega )}}} \right. \kern-\nulldelimiterspace} {(2\Omega )}} = {5 \mathord{\left/ {\vphantom {5 {36}}} \right. \kern-\nulldelimiterspace} {36}}$. As a result, there should be noticeable distortions both in the spectrum and waveform of the transmitted photon. These distortions depend on the initial vibration phase of the absorber as follows from Eqs. (21) and (22) and illustrated in Fig. 4. The distortions are largest at $\vartheta = 0$ and smallest at $\vartheta = {\pi \mathord{\left/ {\vphantom {\pi 2}} \right. \kern-\nulldelimiterspace} 2}$. While the spectral distortion of the photon field is rather modest (Fig. 4a) due to the small residual resonant absorption in the transparency spectral window, the relatively large amplitude modulation of the photon waveform (Fig. 4b,c) shows that the resonant dispersion remains significant for these parameter values.

One can see in Fig. 4b,c that the amplitude modulation in the SMS photon waveform is noticeably less than the modulation of the RMS photon. This is due to the fact that only a part of the spectrum of the SMS photon falls into resonance with the nuclear transition, since the spectral width of SMS is three times larger than the spectral width of the absorber, whereas the spectral width of the absorber is equal to that of RMS. The specified difference in the value of modulation of the transmitted photon waveform is confirmed by the estimation based on Eq. (24) below.

**Figure 5:**
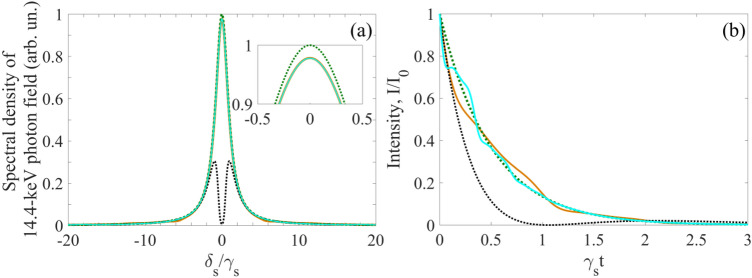
(Color online) Spectrum **(a)** and waveform **(b)** of the Lorentzian single-photon wave packet transmitted through the vibrating ^57^Fe absorber under the conditions of Fig. 4 in the laboratory reference frame for SMS ($\gamma_{S} = 3\gamma_{21}$, brown lines) and for RMS ($\gamma_{S} = \gamma_{21}$, cyan lines) when averaging over the initial vibration phase of the absorber is implemented. All other parameters are the same as in Fig. 4. The brown and cyan curves in **(a)** practically indistinguishable at this scale.

It should be noted that if the absorber vibration frequency is not a multiple of the SR pulse repetition rate, the averaging over $\vartheta$ takes place, which corresponds to the random-phase regime of AIT. In this case, the interference effects in the photon propagation disappear, which leads to a smoothing of both the spectrum and waveform of the transmitted photon (Fig. 5). Deviations in the spectrum and waveform of the transmitted photon are also reduced compared to the incident photon.

As illustrated in Fig. 4 and Fig. 5, the imperfection of transparency is manifested by a reduction in the total transmitted energy due to residual absorption at the resonant nuclear transition and primarily in the regular amplitude modulation of the transmitted photon waveform with the frequency of vibration due to residual resonant dispersion. Therefore, the maximum modulus of deviation of the transmitted photon waveform from the incident photon waveform is a sensitive quantitative characteristic of the “quality” of AIT. In the phase-locked regime, it is a function of the vibration frequency, optical depth, and initial vibration phase,

24 $$D\left( {\Omega ,\,T_{M} ,\,\vartheta } \right) = \frac{{\max \left| {I_{out} (t) - I_{in} (t)} \right|}}{{I_{0} }} ,$$

where $I_{out} (t) = \frac{c}{8\pi }\left| {E^{\prime}_{out} (t)} \right|^{2}$ can be calculated via Eq. (12) or Eq. (16), whereas $I_{in} (t) = \frac{c}{8\pi }\left| {E^{\prime}_{S} (t,z^{\prime} = 0)} \right|^{2}$ can be calculated via Eq. (5) or Eq. (17). The deviation function (Eq. 24) can also serve as a measure of AIT for a single-photon wave packet. Let us estimate it for the above parameter values using Eq. (16). The transmitted field in the vibrating reference frame (Eq. 14) can be represented in the form

25 $${E^{\prime}_{out}}(t) = {E_0}\theta \left( t \right){e^{ - {T_e}/2}}{e^{ - \left( {i{\omega _{SC}} + {\gamma _S}} \right)t + i\varphi }}\sum\limits_{n = - \infty }^\infty {{J_n}\left( p \right){e^{in\left( {\Omega t + \vartheta } \right)}}\left\{ {1 - \int\limits_0^{\sqrt {2{T_M}{\gamma _{21}}t} } {{J_1}\left( x \right){e^{ - \frac{{{\gamma _{21}} - {\gamma _S} + i\left( {n\Omega + {\omega _{21}} - {\omega _{SC}}} \right)}}{{2{T_M}{\gamma _{21}}}}{x^2}}}dx} } \right\}},$$

where the Jacobi-Anger expansion was used. It is a superposition of the incident field (the first term in curly brackets) and the resonant coherently forward-scattered field produced by the nuclear polarization induced by the sidebands (the second term in curly brackets). With taking (Eq. 25) into account, the deviation function (Eq. 24) is the normalized intensity of the coherently forward scattered field plus its interference with the incident field. Thus, the smaller the scattered field, the better AIT and the smaller the deviation function. In the case of AIT (Eq. 7) when $\omega_{SC} = \omega_{21}$ and $p = p_{i}$ in the first order by ${{\gamma_{S} } \mathord{\left/ {\vphantom {{\gamma_{S} } {\Omega \ll 1}}} \right. \kern-\nulldelimiterspace} {\Omega \ll 1}}$, $\gamma_{21} /\Omega \ll 1$, and ${{T_{M} \gamma_{21} } \mathord{\left/ {\vphantom {{T_{M} \gamma_{21} } {\left( {2\Omega } \right)}}} \right. \kern-\nulldelimiterspace} {\left( {2\Omega } \right)}} \ll 1$, Eq. (25) can be integrated and reduced to the form

26 $$\begin{gathered} E^{\prime}_{out} \left( t \right) \approx E_{0} \theta \left( t \right)e^{{ - {{T_{e} } \mathord{\left/ {\vphantom {{T_{e} } 2}} \right. \kern-\nulldelimiterspace} 2}}} e^{{ - \left( {i\omega_{SC} + \gamma_{S} } \right)t + i\varphi }} \sum\limits_{\begin{subarray}{l} n = - \infty \\ \,\,n \ne 0 \end{subarray} }^{\infty } {J_{n} \left( {p_{i} } \right)e^{{in\left( {\Omega t + \vartheta } \right)}} \left[ {1 - \frac{{{{T_{M} \gamma_{21} } \mathord{\left/ {\vphantom {{T_{M} \gamma_{21} } {\left( {2\Omega } \right)}}} \right. \kern-\nulldelimiterspace} {\left( {2\Omega } \right)}}}}{{{{\Delta \gamma } \mathord{\left/ {\vphantom {{\Delta \gamma } {\Omega + in}}} \right. \kern-\nulldelimiterspace} {\Omega + in}}}}} \right]} + \frac{{T_{M} \gamma_{21} }}{\Omega }E_{0} \theta \left( t \right)e^{{ - {{T_{e} } \mathord{\left/ {\vphantom {{T_{e} } 2}} \right. \kern-\nulldelimiterspace} 2}}} e^{{ - \left( {i\omega_{21} + \gamma_{21} } \right)t + i\varphi }} \frac{{J_{1} \left( {\sqrt {2T_{M} \gamma_{21} t} } \right)}}{{\sqrt {2T_{M} \gamma_{21} t} }}\sum\limits_{\begin{subarray}{l} n = - \infty \\ \,\,n \ne 0 \end{subarray} }^{\infty } {\frac{{J_{n} \left( {p_{i} } \right)e^{in\vartheta } }}{{{{\Delta \gamma } \mathord{\left/ {\vphantom {{\Delta \gamma } {\Omega + in}}} \right. \kern-\nulldelimiterspace} {\Omega + in}}}}} , \hfill \\ \end{gathered}$$

where $\Delta \gamma = \gamma_{21} - \gamma_{S}$. The first sum in Eq. (26) is a superposition of the transmitted sidebands at their peak frequencies, $n\Omega$, transformed according to Eq. (22) due to their interaction with the wings of the nuclear transition. The second sum is a superposition of the transmitted wings of sidebands at the nuclear resonant frequency, $\omega_{21}$. Calculating the intensity, $I_{out} (t)$, one obtains

27 $$D\left( {\Omega ,\,T_{M} ,\,\vartheta } \right) \approx \frac{{T_{M} \gamma_{21} }}{\Omega }\max \left| {e^{{ - 2\gamma_{S} t}} \sum\limits_{\begin{subarray}{l} n = - \infty , \\ n \ne 0 \end{subarray} }^{\infty } {\frac{{J_{n} \left( {p_{1} } \right)}}{n}\left\{ {\sin \left[ {p_{1} \sin \left( {\Omega t + \vartheta } \right) - n\left( {\Omega t + \vartheta } \right)} \right] - e^{ - t\Delta \gamma } \frac{{2J_{1} \left( {\sqrt {2T_{M} \gamma_{21} t} } \right)}}{{\sqrt {2T_{M} \gamma_{21} t} }}\sin \left[ {p_{1} \sin \left( {\Omega t + \vartheta } \right) - n\vartheta } \right]} \right\}} } \right| .$$

As follows from Eq. (27), the waveform of the transmitted photon acquires an amplitude modulation with frequency of the absorber vibration. Similar to Eq. (20), at $\vartheta = {\pi \mathord{\left/ {\vphantom {\pi 2}} \right. \kern-\nulldelimiterspace} 2}$ the second term in Eq. (27) is negligible and the deviation function is reduced to

28 $$D\left( {\Omega ,\,T_{M} ,\,{\pi \mathord{\left/ {\vphantom {\pi 2}} \right. \kern-\nulldelimiterspace} 2}} \right) \approx \frac{{T_{M} \gamma_{21} }}{\Omega }\max \left| {e^{{ - 2\gamma_{S} t}} \sum\limits_{n = - \infty ,n \ne 0}^{\infty } {\frac{{J_{n} \left( {p_{1} } \right)}}{n}\sin \left[ {p_{1} \sin \left( {\Omega t + \frac{\pi }{2}} \right) - n\left( {\Omega t + \frac{\pi }{2}} \right)} \right]} } \right|.$$

**Figure 6:**
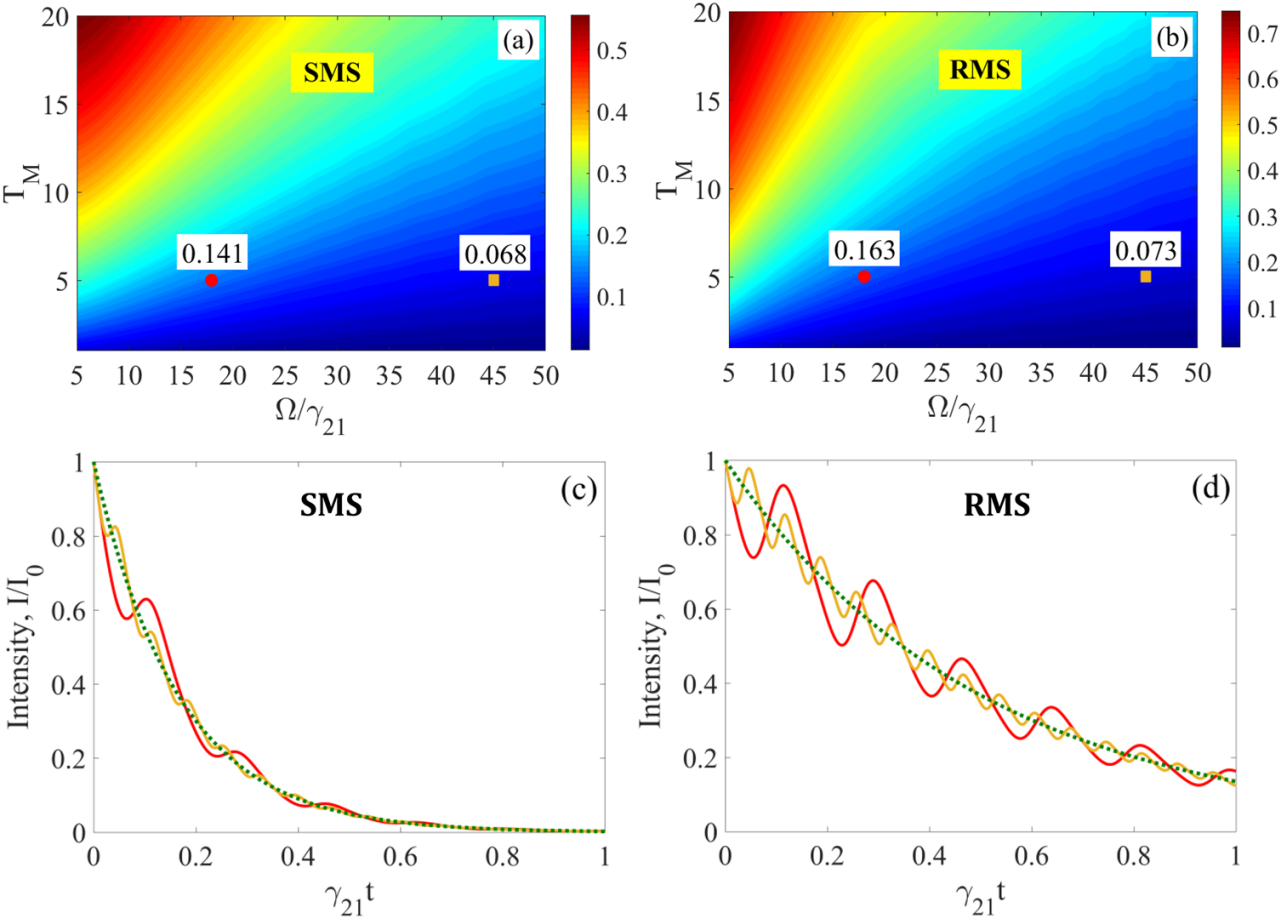
(Color online) Deviation function $D\left( {\Omega ,\,T_{M} ,\,{\pi \mathord{\left/ {\vphantom {\pi 2}} \right. \kern-\nulldelimiterspace} 2}} \right)$ plotted according to Eqs. (24) and (12) at $p = p_{1}$ for SMS with $\gamma_{S} = 3\gamma_{21}$ in **(a)**, and for RMS with $\gamma_{S} = \gamma_{21}$ in **(b)**. In both panels $\omega_{SC} = \omega_{21}$ and $T_{e} = 0$. The red circles indicate the deviation function values corresponding to ${{T_{M} \gamma_{21} } \mathord{\left/ {\vphantom {{T_{M} \gamma_{21} } {(2\Omega )}}} \right. \kern-\nulldelimiterspace} {(2\Omega )}} = {5 \mathord{\left/ {\vphantom {5 {36}}} \right. \kern-\nulldelimiterspace} {36}}$, while ${{\gamma_{S} } \mathord{\left/ {\vphantom {{\gamma_{S} } \Omega }} \right. \kern-\nulldelimiterspace} \Omega } = {1 \mathord{\left/ {\vphantom {1 6}} \right. \kern-\nulldelimiterspace} 6}$ in **(a)** and ${{\gamma_{S} } \mathord{\left/ {\vphantom {{\gamma_{S} } \Omega }} \right. \kern-\nulldelimiterspace} \Omega } = {1 \mathord{\left/ {\vphantom {1 {18}}} \right. \kern-\nulldelimiterspace} {18}}$ in **(b)** as in Fig. 4. The orange squares indicate the deviation function values corresponding to ${{T_{M} \gamma_{21} } \mathord{\left/ {\vphantom {{T_{M} \gamma_{21} } {(2\Omega )}}} \right. \kern-\nulldelimiterspace} {(2\Omega )}} = {1 \mathord{\left/ {\vphantom {1 {18}}} \right. \kern-\nulldelimiterspace} {18}}$, while ${{\gamma_{S} } \mathord{\left/ {\vphantom {{\gamma_{S} } \Omega }} \right. \kern-\nulldelimiterspace} \Omega } = {1 \mathord{\left/ {\vphantom {1 {15}}} \right. \kern-\nulldelimiterspace} {15}}$ in **(a)** and ${{\gamma_{S} } \mathord{\left/ {\vphantom {{\gamma_{S} } \Omega }} \right. \kern-\nulldelimiterspace} \Omega } = {1 \mathord{\left/ {\vphantom {1 {45}}} \right. \kern-\nulldelimiterspace} {45}}$ in **(b)**. The normalized photon waveforms corresponding to the red circles are shown in **(c)** and **(d)** as the red curves, and those corresponding to the orange squares are the orange curves. The incident photon waveform is represented by the green dotted curves.

The deviation function Eq. (24) at $\vartheta = {\pi \mathord{\left/ {\vphantom {\pi 2}} \right. \kern-\nulldelimiterspace} 2}$ for SMS with $\gamma_{S} = 3\gamma_{21}$ and for RMS with $\gamma_{S} = \gamma_{21}$ is plotted in Fig. 6a,b. In the domain where AIT conditions (Eq. 23) are near the boundary of feasibility (the red circles in Fig. 6a,b), the deviation function calculated by Eq. (28) is $D \approx 0.149$ for SMS and $D \approx 0.181$ for RMS, which are in a good agreement with the numerical estimate by Eq. (24). The deviation function (Eq. 28) for SMS photon is smaller than for RMS photon due to the larger exponent in the term $e^{{ - 2\gamma_{S} t}}$. In the limit where conditions (Eq. 23) are well satisfied, the transmitted photon waveform approaches the incident photon waveform and practically does not depend on the linewidth of the photon source as illustrated by the yellow squares in Fig. 6a,b (Eq. (28) gives $D \approx 0.071$ for SMS and $D \approx 0.077$ for RMS). For a given width of the absorber spectral line, the AIT conditions (Eq. 23) can be met better via decreasing the absorber optical depth or via increasing the vibration frequency as shown in Fig. 6. An increase in the vibration frequency leads to a smaller-scale amplitude modulation in the waveform of the transmitted photon approaching the incident photon waveform (Fig. 6c,d).

In the random-phase regime where the absorber vibration phase is not locked to the front of the single-photon wave packet, the amplitude modulation in the transmitted photon waveform is averaged over the initial phase of vibration and the waveform is smoothed (Fig. 5b). The deviation function for the averaged photon waveform can be determined similar to Eq. (24) as

29 $$\left\langle {D\left( {\Omega ,\,T_{M} } \right)} \right\rangle_{\vartheta } = \frac{{\max \left| {\left\langle {I_{out} (t)} \right\rangle_{\vartheta } - I_{in} (t)} \right|}}{{I_{0} }}.$$

At $p = p_{1}$ and $\omega_{SC} = \omega_{21}$ in the first order by ${{\gamma_{S} } \mathord{\left/ {\vphantom {{\gamma_{S} } {\Omega \ll 1}}} \right. \kern-\nulldelimiterspace} {\Omega \ll 1}}$, $\gamma_{21} /\Omega \ll 1$, and ${{T_{M} \gamma_{21} } \mathord{\left/ {\vphantom {{T_{M} \gamma_{21} } {\left( {2\Omega } \right)}}} \right. \kern-\nulldelimiterspace} {\left( {2\Omega } \right)}} \ll 1$, it can be estimated from Eq. (27) as

30 $$\left\langle {D\left( {\Omega ,\,T_{M} } \right)} \right\rangle_{\vartheta } \approx \frac{{T_{M} \gamma_{21} }}{\Omega }\max \left| {e^{{ - (\gamma_{21} + \gamma_{S} )t}} \frac{{2J_{1} \left( {\sqrt {2T_{M} \gamma_{21} t} } \right)}}{{\sqrt {2T_{M} \gamma_{21} t} }}\sum\limits_{n = - \infty ,n \ne 0}^{\infty } {\frac{{J_{n}^{2} \left( {p_{1} } \right)}}{n}\sin \left( {n\Omega t} \right)} } \right| .$$

The maximum of modulus in Eq. (30) is achieved at $t = {\pi \mathord{\left/ {\vphantom {\pi {(2\Omega )}}} \right. \kern-\nulldelimiterspace} {(2\Omega )}}$. For the case plotted in Fig. 5 and for the red circles in Fig. 6, one has $\left\langle D \right\rangle_{\vartheta } \approx 0.09$ for SMS and $\left\langle D \right\rangle_{\vartheta } \approx 0.11$ for RMS. For yellow squares in Fig. 6 the deviation function (Eq. 30) is $\left\langle D \right\rangle_{\vartheta } \approx 0.05$ for both sources. Similar to the case of phase-locked regime, the deviation function (Eq. 30) for SMS photon is less than for RMS photon due to the term $e^{{ - (\gamma_{21} + \gamma_{S} )t}}$ corresponding to broader incident field spectrum. In the domain where the AIT conditions (Eq. 23) are perfectly met, the deviation function for the averaged photon waveform in both cases of SMS and RMS approaches the value $\left\langle {D\left( {\Omega ,\,T_{M} } \right)} \right\rangle_{\vartheta } \approx {{T_{M} \gamma_{21} } \mathord{\left/ {\vphantom {{T_{M} \gamma_{21} } {(2\Omega )}}} \right. \kern-\nulldelimiterspace} {(2\Omega )}}$.

As follows from Eqs. (27) and (30), the vibration with high enough frequency allows one to induce a sufficient degree of transparency for SMS photons in absorber with high optical depth. For example, vibration of the absorber with available frequency of 24 GHz^58^ can provide AIT with the waveform deviation of $D < 10^{ - 2}$ for the optical depth up to $T_{M} \approx 100$.

Thus, in the phase-locked regime, the transmitted photon acquires regular amplitude modulation of its waveform as a result of the interference effects during the photon propagation. As a result, AIT in the phase-locked regime requires stricter fulfillment of conditions (Eq. 23) as compared to the random-phase regime. Amplitude modulation may be reduced by the proper choice of the initial vibration phase. It can also be suppressed by increasing the vibration frequency. It is worth noting that due to the micron-sized beam cross-section, SMS allows to use higher vibration frequency compared to RMS without degrading the uniformity of the nuclear vibration amplitude across the photon beam, necessary for AIT. Moreover, under the same conditions SMS allows achieving higher quality of AIT in the phase-locked regime compared to RMS due to the shorter pulse duration.

## AIT in the phase-locked regime for Lorentz-squared photon

If the ^57^FeBO_3_ crystal is heated in an external magnetic field to the Néel temperature, the multiline Mössbauer radiation produced by SMS can collapse into a single spectral line similar to the spectral line of 14.4-keV radiation emitted by the ^57^Co radioactive source^47–53^. The produced quasi-monochromatic radiation is the result of several mechanisms of interaction between the SR pulse and a lattice of oriented ^57^Fe nuclear spins. Therefore, it has a non-Lorentzian spectral shape, which is determined by the temperature of the ^57^FeBO_3_ crystal, its angular position relative to the incident SR beam near the Bragg angle, and the applied magnetic fields^47–53^. Depending on the conditions, the wings of the SMS spectral line can fall faster or slower than the wings of the RMS Lorentzian line. In all implemented cases, the measured SMS spectral line at the Néel temperature was several times wider than the RMS line at room temperature. Correspondingly, in contrast to RMS, the single photon waveform has gradually increasing front and shorter duration^47–53^, which can be more favorable for some applications including quantum information processing.

Under certain experimentally realized conditions, the measured SMS spectral line can be described via a superposition of fields from two close homogeneously broadened nuclear transitions^47–50^,

31 $$\tilde{E}_{S} (\delta_{S} ) = \tilde{E}_{S} (0)\left\{ {\frac{1}{{1 + {{i(\delta_{S} - {\Delta \mathord{\left/ {\vphantom {\Delta 2}} \right. \kern-\nulldelimiterspace} 2})} \mathord{\left/ {\vphantom {{i(\delta_{S} - {\Delta \mathord{\left/ {\vphantom {\Delta 2}} \right. \kern-\nulldelimiterspace} 2})} {\gamma_{S} }}} \right. \kern-\nulldelimiterspace} {\gamma_{S} }}}} - \frac{1}{{1 + {{i(\delta_{S} + {\Delta \mathord{\left/ {\vphantom {\Delta 2}} \right. \kern-\nulldelimiterspace} 2})} \mathord{\left/ {\vphantom {{i(\delta_{S} + {\Delta \mathord{\left/ {\vphantom {\Delta 2}} \right. \kern-\nulldelimiterspace} 2})} {\gamma_{S} }}} \right. \kern-\nulldelimiterspace} {\gamma_{S} }}}}} \right\},$$

where $\delta_{S} = \omega_{SC} - \omega_{S}$, $\omega_{SC} = {{\left( {\omega_{1} + \omega_{2} } \right)} \mathord{\left/ {\vphantom {{\left( {\omega_{1} + \omega_{2} } \right)} 2}} \right. \kern-\nulldelimiterspace} 2}$, and $\Delta = \omega_{2} - \omega_{1}$ is the difference between the central frequencies, $\omega_{1}$ and $\omega_{2}$, of the corresponding Lorentzian lines ($\omega_{2} > \omega_{1}$ is assumed for certainty), which can be controlled by the experimental conditions. The measured amplitude spectrum of the field (31) is then

32 $$\left| {\tilde{E}_{S} (\delta_{S} )} \right|^{2} = \left| {\tilde{E}_{S} (0)} \right|^{2} \frac{{(\Delta /\gamma_{S} )^{2} }}{{\left[ {1 + (\delta_{S} - \Delta /2)^{2} /\gamma_{S}^{2} } \right]\left[ {1 + (\delta_{S} + \Delta /2)^{2} /\gamma_{S}^{2} } \right]}}\mathop \approx \limits^{{\Delta \ll \gamma_{S} }} \left| {\tilde{E}_{S} (0)} \right|^{2} \frac{{(\Delta /\gamma_{S} )^{2} }}{{\left( {1 + (\delta_{S} /\gamma_{S} )^{2} } \right)^{2} }}.$$

According to Eq. (32), at $\Delta \ll \gamma_{S}$ the resulting spectral line of SMS constitutes a pseudo-single line with the Lorentz-squared shape. Its amplitude is $\left( {\Delta /\gamma_{S} } \right)^{2}$ times less than the amplitude of the single Lorentz line (Eq. 18). However, at certain conditions the amplitude of the Lorentz-squared line can be comparable to the amplitude of a single Lorentz line after extraction from the sextet^47–50^. At the same time, the halfwidth at half-maximum of the Lorentz-squared line is $\gamma_{LS} \approx 0.64\gamma_{S}$, and its wings fall faster than the wings of the Lorentz line with halfwidth $\gamma_{S}$. However, as follows from^47–50^, each Lorentzian component of the Lorentz-squared doublet at the Néel temperature is more broadened, $2\gamma_{S} \approx 4.7\Gamma_{0}$, than the Lorentz lines in the sextet at room temperature, $2\gamma_{S} \approx 2.9\Gamma_{0}$. As a result, the measured width of the Lorentz-squared line is approximately the same as the width of the Lorentzian line discussed in the previous section, $2\gamma_{LS} \approx 3\Gamma_{0}$.

**Figure 7:**
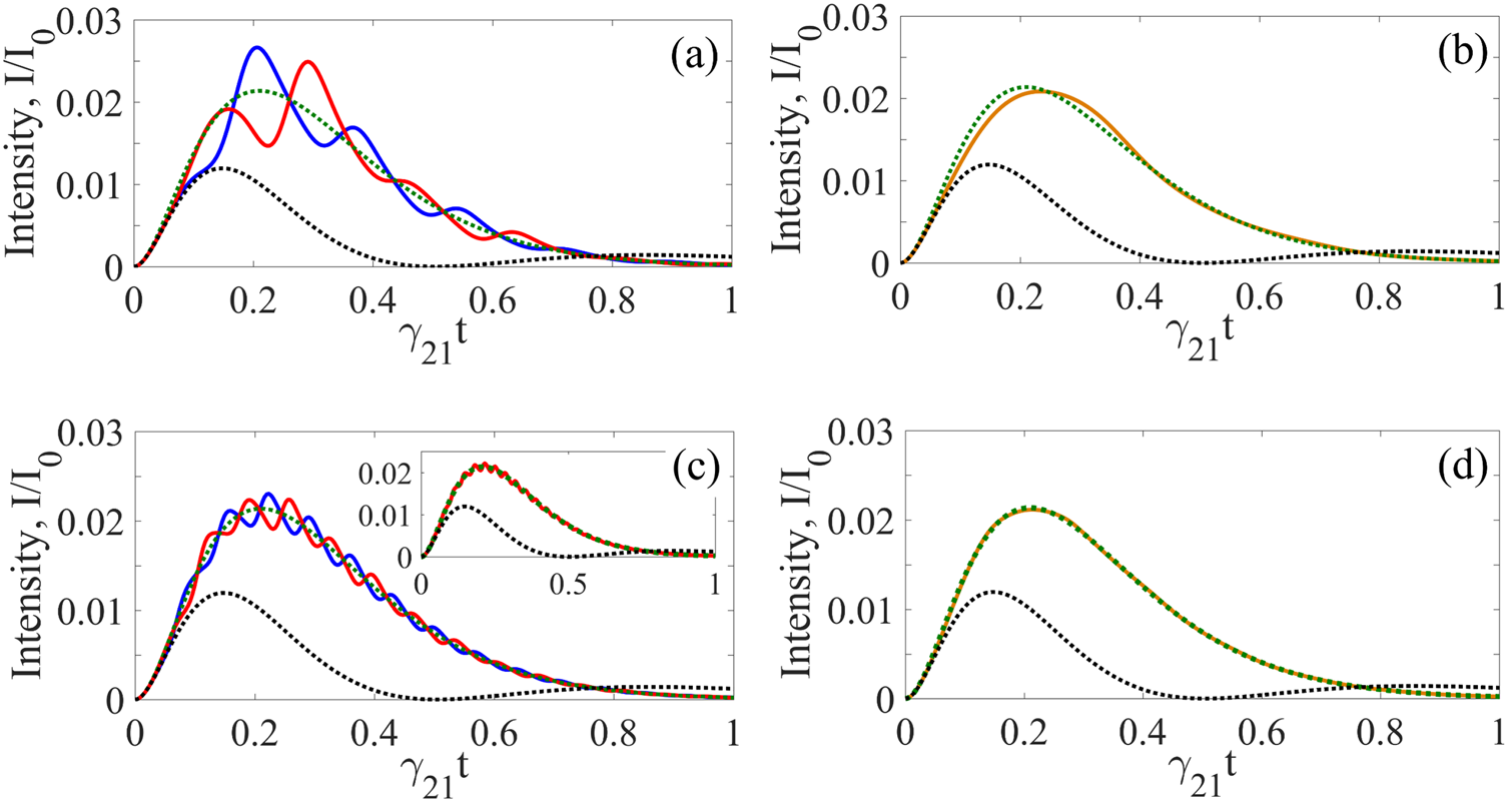
(Color online) Waveform of the SMS Lorentz-squared photon [the green dotted curves, Eq. (34)] transmitted through the motionless absorber (dotted black curves) and the vibrating absorber at $\vartheta = 0$ (in blue), $\vartheta = {\pi \mathord{\left/ {\vphantom {\pi 2}} \right. \kern-\nulldelimiterspace} 2}$ (in red), and averaged over $\vartheta$ (in brown). All are plotted as the normalized function ${{I_{out} (t)} \mathord{\left/ {\vphantom {{I_{out} (t)} {I_{0} }}} \right. \kern-\nulldelimiterspace} {I_{0} }}$ [where $I_{0} = {{cE_{0}^{2} } \mathord{\left/ {\vphantom {{cE_{0}^{2} } {(8\pi )}}} \right. \kern-\nulldelimiterspace} {(8\pi )}}$] according to Eq. (12) with $\tilde{E}_{S} (\delta_{S} )$ from Eq. (31) for the same as in Fig. 3 vibrating absorber: $T_{e} = 0$, $T_{M} = 5$, $\gamma_{21} = {{\Gamma_{0} } \mathord{\left/ {\vphantom {{\Gamma_{0} } 2}} \right. \kern-\nulldelimiterspace} 2}$, $R = R_{1}$. The vibration frequency is ${\Omega \mathord{\left/ {\vphantom {\Omega {\gamma_{21} }}} \right. \kern-\nulldelimiterspace} {\gamma_{21} }} = 18$ (i.e., ${\Omega \mathord{\left/ {\vphantom {\Omega {(2\pi )}}} \right. \kern-\nulldelimiterspace} {(2\pi )}} \approx 10.2{\text{ MHz}}$) for **(a)** and **(b)**, ${\Omega \mathord{\left/ {\vphantom {\Omega {\gamma_{21} }}} \right. \kern-\nulldelimiterspace} {\gamma_{21} }} = 45$ (i.e., ${\Omega \mathord{\left/ {\vphantom {\Omega {(2\pi )}}} \right. \kern-\nulldelimiterspace} {(2\pi )}} \approx 25.5{\text{ MHz}}$) for **(c)** and **(d)** and ${\Omega \mathord{\left/ {\vphantom {\Omega {\gamma_{21} }}} \right. \kern-\nulldelimiterspace} {\gamma_{21} }} = 90$ (i.e., ${\Omega \mathord{\left/ {\vphantom {\Omega {(2\pi )}}} \right. \kern-\nulldelimiterspace} {(2\pi )}} \approx 51{\text{ MHz}}$) for inset in **(c)**. Each component of the spectral doublet (Eq. 31) with splitting $\Delta = 0.4\gamma_{S}$, has a width ${{\gamma_{S} } \mathord{\left/ {\vphantom {{\gamma_{S} } {\gamma_{21} }}} \right. \kern-\nulldelimiterspace} {\gamma_{21} }} = 4.7$ (see text), so that the resulting Lorentz-squared SMS line has a width $\gamma_{LS} \approx 3\gamma_{21}$, like the Lorentz line in Fig. 5.

The inverse Fourier transform of Eq. (31) gives the time dependence of the electric field of a single-photon wave packet with Lorentz-squared spectrum before the absorber in the form of beats,

33 $$E_{S} (t,z \leqslant 0) = E_{0} \theta \left( \tau \right)e^{{ - \gamma_{S} \tau + i\varphi }} \left\{ {e^{{ - i\omega_{1} \tau }} - e^{{ - i\omega_{2} \tau }} } \right\} = E_{0} \theta \left( \tau \right)e^{{ - (i\omega_{SC} + \gamma_{S} )\tau + i\varphi }} 2i\sin \left( {\frac{\Delta }{2}\tau } \right),$$

where $\tau = t - z/c$. The corresponding waveform of a photon incident on the absorber, at $z = 0$, has a gradually increasing front edge determined by Δ (Fig. 7),

34 $$I_{in} (t) = 4\theta \left( t \right)I_{0} \sin^{2} \left( {\frac{\Delta }{2}t} \right)e^{{ - 2\gamma_{S} t}} ,$$

where $I_{0} = cE_{0}^{2} /(8\pi )$. For a given value of $\gamma_{S}$, the waveform duration of the Lorentz-squared photon, evaluated as FWHM, is determined by $\Delta$ and approaches the value of about ${{1.7} \mathord{\left/ {\vphantom {{1.7} {\gamma_{S} }}} \right. \kern-\nulldelimiterspace} {\gamma_{S} }} \approx {{0.36} \mathord{\left/ {\vphantom {{0.36} {\gamma_{21} }}} \right. \kern-\nulldelimiterspace} {\gamma_{21} }}$ at $\Delta \ll \gamma_{S}$, which is close to the FWHM duration of the Lorentzian single-photon wave packet from RMS (about ${{0.35} \mathord{\left/ {\vphantom {{0.35} {\gamma_{21} }}} \right. \kern-\nulldelimiterspace} {\gamma_{21} }}$).

The propagation of a single-photon wave packet (Eq. 33) with a pseudo-single-line spectrum (Eq. 31) through the vibrating absorber in the phase-locked regime can be described by Eqs. (1)–(6) and (8)–(13) as a generalization of formulae Eqs. (17)–(22), (25), and (26) to the case of two close single-line Lorentzian spectra. For example, in the reference frame of the vibrating absorber, the incident field looks as a comb (Eq. 6) (see also Fig. 2) of pseudo-single lines centered at frequencies $\omega_{SC} + n\Omega$, $n \in {\mathbb{Z}}$, governed by the equation

35 $$\tilde{E}_{S}^{(n)} (\delta_{S} )\, = \tilde{E}_{S} (0)J_{ - n} (p)e^{ - in\vartheta } \left\{ {\frac{1}{{1 + i{{\left( {\delta_{S} + n\Omega - {\Delta \mathord{\left/ {\vphantom {\Delta 2}} \right. \kern-\nulldelimiterspace} 2}} \right)} \mathord{\left/ {\vphantom {{\left( {\delta_{S} + n\Omega - {\Delta \mathord{\left/ {\vphantom {\Delta 2}} \right. \kern-\nulldelimiterspace} 2}} \right)} {\gamma_{S} }}} \right. \kern-\nulldelimiterspace} {\gamma_{S} }}}}\, - \frac{1}{{1 + i{{\left( {\delta_{S} + n\Omega + {\Delta \mathord{\left/ {\vphantom {\Delta 2}} \right. \kern-\nulldelimiterspace} 2}} \right)} \mathord{\left/ {\vphantom {{\left( {\delta_{S} + n\Omega + {\Delta \mathord{\left/ {\vphantom {\Delta 2}} \right. \kern-\nulldelimiterspace} 2}} \right)} {\gamma_{S} }}} \right. \kern-\nulldelimiterspace} {\gamma_{S} }}}}} \right\} .$$

At the exit from the absorber in the case (Eq. 7) of AIT, the resonant part of the spectrum (at frequency $\omega_{S} = \omega_{SC} = \omega_{21}$) in the vibrating reference frame in the case $\Omega \gg \{ \gamma_{S} ,\gamma_{21} \} \gg \Delta$ is described by Eq. (19), where

36 $${\tilde E^{\prime}_S}(0,0) \approx {\tilde E_S}(0)\frac{{2\Delta }}{{{\gamma _S}}}{\left( {\frac{{{\gamma _S}}}{\Omega }} \right)^2}\sum\limits_{n = 1}^\infty {\left\{ {\frac{{{J_{2n - 1}}({p_i})}}{{{{(2n - 1)}^2}}}\,\sin \left[ {(2n - 1)\vartheta } \right] - i\frac{{{J_{2n}}({p_i})}}{{4{n^2}}}\,\cos (2n\vartheta )} \right\}} .$$

Similar to (Eq. 20), the total resonant part of the sideband wings depends on the initial phase of the absorber vibration. As follows from Eq. (36), at $p = p_{1}$ unlike (Eq. 20), there is no value of $\vartheta$, at which the resonant field equals zero. In addition, oppositely to (Eq. 20), the minimum and maximum values of $\left| {\tilde{E^{\prime}_{S}} (0,0)} \right|$ are achieved approximately at $\vartheta = k\pi$ and $\vartheta = {{(2k + 1)\pi } \mathord{\left/ {\vphantom {{(2k + 1)\pi } 2}} \right. \kern-\nulldelimiterspace} 2}$, $k \in {\mathbb{Z}}$, respectively, due to the difference in spectral phases of the Lorentz-squared and Lorentz spectra. Comparing Eqs. (36) and (32) with Eqs. (20) and (18), one can conclude that the maximum value of the resonant part of the field in case of the Lorentz-squared spectral line has the second order of smallness by ${{\gamma_{S} } \mathord{\left/ {\vphantom {{\gamma_{S} } \Omega }} \right. \kern-\nulldelimiterspace} \Omega }$, compared to the first order of smallness in the case of the Lorentz line, due to the rapidly falling wings of the Lorentz-squared line. As a result, in the first order of smallness by ${{\gamma_{S} } \mathord{\left/ {\vphantom {{\gamma_{S} } \Omega }} \right. \kern-\nulldelimiterspace} \Omega }$, only the wings of the nuclear transition line at the central frequencies of sidebands, $\omega_{S} = \omega_{SC} + m\Omega$, $m = 1,2,...$, cause the distortions in the spectral-temporal characteristics of the transmitted Lorentz-squared photon,

37 $$\tilde{E^{\prime}_{S}} (m\Omega ,L) \approx e^{{{{ - T_{e} } \mathord{\left/ {\vphantom {{ - T_{e} } 2}} \right. \kern-\nulldelimiterspace} 2}}} \tilde{E}_{S} (0)\,\frac{i\Delta }{{\gamma_{S} }}\left\{ {1 - i\frac{{T_{M} \gamma_{21} }}{2m\Omega }} \right\}J_{ - m} (p_{i} )e^{ - im\vartheta }.$$

This dependence is the same as described by Eq. (22).

The alterations of the wave packet (Eq. 33) at the exit from the vibrating absorber can be described either by Eqs. (10)–(12) with substituting (Eq. 35) or by Eqs. (14)–(16) with substituting (Eq. 33). In the latter case, Eq. (25) becomes

38 $$E^{\prime}_{out} (t) = E^{\prime}_{out(1)} (t) - E^{\prime}_{out(2)} (t) ,$$

where

39 $$E^{\prime}_{out(1)} (t) = E_{0} \theta \left( t \right)e^{{ - T_{e} /2}} e^{{ - \left( {i\omega_{1} + \gamma_{S} } \right)t + i\varphi }} \sum\limits_{n = - \infty }^{\infty } {J_{n} \left( p \right)e^{{in\left( {\Omega t + \vartheta } \right)}} \left\{ {1 - \int\limits_{0}^{{\sqrt {2T_{M} \gamma_{21} t} }} {J_{1} \left( x \right)e^{{ - \frac{{\gamma_{21} - \gamma_{S} }}{{2T_{M} \gamma_{21} }}x^{2} - i\frac{{n\Omega + \omega_{21} - \omega_{1} }}{{2T_{M} \gamma_{21} }}x^{2} }} dx} } \right\}} ,$$

and

40 $$E^{\prime}_{out(2)} (t) = E_{0} \theta \left( t \right)e^{{ - T_{e} /2}} e^{{ - \left( {i\omega_{2} + \gamma_{S} } \right)t + i\varphi }} \sum\limits_{n = - \infty }^{\infty } {J_{n} \left( p \right)e^{{in\left( {\Omega t + \vartheta } \right)}} \left\{ {1 - \int\limits_{0}^{{\sqrt {2T_{M} \gamma_{21} t} }} {J_{1} \left( x \right)e^{{ - \frac{{\gamma_{21} - \gamma_{S} }}{{2T_{M} \gamma_{21} }}x^{2} - i\frac{{n\Omega + \omega_{21} - \omega_{2} }}{{2T_{M} \gamma_{21} }}x^{2} }} dx} } \right\}}.$$

In the case of AIT (Eq. 7) when $p = p_{i}$ and $\omega_{SC} = \omega_{21}$ in the first order by $\gamma_{S} /\Omega \ll 1$, $\gamma_{21} /\Omega \ll 1$, and ${{T_{M} \gamma_{21} } \mathord{\left/ {\vphantom {{T_{M} \gamma_{21} } {\left( {2\Omega } \right)}}} \right. \kern-\nulldelimiterspace} {\left( {2\Omega } \right)}} \ll 1$, Eqs. (38)–(40) can be integrated similar to Eq. (25) and reduced to the form similar to (Eq. 26),

41 $${E^{\prime}_{out}}(t) \approx {E_0}\theta \left( t \right){e^{ - {T_e}/2}}{e^{ - (i{\omega _{SC}} + {\gamma _S})t + i\varphi }}2i\sin \left( {\frac{\Delta }{2}t} \right)\sum\limits_{n = - \infty ,n \ne 0}^\infty {{J_n}\left( {{p_i}} \right){e^{in\left( {\Omega t + \vartheta } \right)}}\left[ {1 - \frac{{{T_M}{\gamma _{21}}}}{{2\Omega }}\frac{1}{{{{\Delta \gamma } \mathord{\left/ {\vphantom {{\Delta \gamma } \Omega }} \right. \kern-\nulldelimiterspace} \Omega } + in}}} \right]} ,$$

where the second term is absent due to the small wings of the Lorentz-squared sidebands at the nuclear resonant frequency, $\omega_{21}$. Just that term describes the dependence of the transmitted field amplitude on the initial phase of the absorber vibration. Thus, the deviation function (Eq. 24) for the Lorentz-squared photon in this case is similar to the deviation function for the Lorentzian photon at $p = p_{1}$ and $\vartheta = {\pi \mathord{\left/ {\vphantom {\pi 2}} \right. \kern-\nulldelimiterspace} 2}$ [Eq. (28)], and practically does not depend on the initial phase of the absorber vibration,

42 $$D\left( {\Omega ,\,T_{M} ,\,{\pi \mathord{\left/ {\vphantom {\pi 2}} \right. \kern-\nulldelimiterspace} 2}} \right) \approx \frac{{4T_{M} \gamma_{21} }}{\Omega }\max \left| {\sin^{2} \left( {\frac{\Delta }{2}t} \right)e^{{ - 2\gamma_{S} t}} \sum\limits_{n = - \infty ,n \ne 0}^{\infty } {\frac{{J_{n} \left( {p_{1} } \right)}}{n}\sin \left[ {p_{1} \sin \left( {\Omega t + \frac{\pi }{2}} \right) - n\left( {\Omega t + \frac{\pi }{2}} \right)} \right]} } \right|.$$

This can also be seen in Fig. 7a, where even with poor AIT, a change in the initial vibration phase from $\vartheta = 0$ to $\vartheta = {\pi \mathord{\left/ {\vphantom {\pi 2}} \right. \kern-\nulldelimiterspace} 2}$ changes primarily the relative phase of the regular amplitude modulation in the photon waveform. In this case, the value of deviation function (Eq. 42) relative to the maximum of the incident photon waveform is 0.19. Similar to the Lorentzian photon, in the case of SMS, the distortion of the photon waveform can be reduced by increasing the vibration frequency (Fig. 7c) without breaking the uniformity condition of the nuclei oscillation amplitudes due to the small cross section of the photon beam.

If AIT is implemented in the regime of random vibration phase of the absorber relative to the front of the single-photon wave packet, the deviation function (Eq. 29) for the averaged photon waveform has the second order of smallness by ${{T_{M} \gamma_{21} } \mathord{\left/ {\vphantom {{T_{M} \gamma_{21} } \Omega }} \right. \kern-\nulldelimiterspace} \Omega }$.

43 $$\left\langle {D\left( {\Omega ,\,T_{M} } \right)} \right\rangle_{\vartheta } \approx C_{LS} \left( {\frac{{T_{M} \gamma_{21} }}{\Omega }} \right)^{2},$$

where $C_{LS} \approx 2.4 \times 10^{ - 2}$. This can be seen from Fig. 7b,d, where the transmitted photon waveform and the incident photon waveform almost coincide at the absorber vibration frequency of 25 MHz (Fig. 7d) and are practically indistinguishable at ${\Omega \mathord{\left/ {\vphantom {\Omega {(2\pi )}}} \right. \kern-\nulldelimiterspace} {(2\pi )}} \approx 51{\text{ MHz}}$ in Fig. 7d.

Thus, both in the phase-locked and random-phase regimes of AIT, the deviation function is practically independent of either the width of the SMS Lorentz-squared line or the initial phase of the absorber vibration. It is determined by the value of the absorber vibration frequency relative to its linewidth, multiplied by the optical depth (the second inequality in (Eq. 23)), and is as small as the deviation function for the Lorentz-line photon in the case of the optimal vibration phase. It can be reduced by increasing the vibration frequency, without breaking the requirement of uniformity of the nuclei oscillation amplitudes.

## AIT in the phase-locked regime via dual-tone vibration

As discussed above, the distortions in the spectral-temporal characteristics of the single-photon wave packet decrease with increasing the absorber vibration frequency and approach the characteristics of the incident photon when the AIT conditions (Eq. 23) are perfectly met. However, an increase in the absorber vibration frequency usually destroys the uniformity of the oscillation amplitude of nuclei. In the case of SMS, this can be overcome by a tighter focusing of radiation. At a relatively low vibration frequency (compared to the product of the optical depth by the linewidth of the absorber (Eq. 23)), spectral distortion and amplitude modulation of the transmitted photon waveform can be reduced via using dual-tone vibration, i.e., by adding its first subharmonic to the fundamental frequency^31^. Let's consider the case when the absorbing nuclei perform such a dual-tone oscillation along the direction of the radiation propagation,

44 $$z = z^{\prime} + R_{H} \sin \left( {{{\Omega t} \mathord{\left/ {\vphantom {{\Omega t} 2}} \right. \kern-\nulldelimiterspace} 2} + \vartheta_{H} } \right) + R\sin \left( {\Omega t + \vartheta } \right) ,$$

where similar to (Eq. 4), $R$, $\vartheta$ and $R_{H}$, $\vartheta_{H}$ are the amplitudes and the initial phases of the absorber vibration at the fundamental frequency, $\Omega$, and its sub-harmonic, ${\Omega \mathord{\left/ {\vphantom {\Omega 2}} \right. \kern-\nulldelimiterspace} 2}$, respectively. All the above consideration can be generalized to this case. In particular, substitution of Eq. (44) into Eq. (1) yields a dual-tone modulated field in the vibrating reference frame, similar to Eqs. (5). The corresponding spectrum at the absorber entrance can be written in the form (Eq. 6) as a superposition of the spectral lines of the incident field (Eq. 3), shifted from the carrier frequency by a frequency multiple of half the fundamental vibration frequency,

45 $$\tilde{E^{\prime}_{S}} (\delta_{S} ,0) = \sum\limits_{n = - \infty }^{\infty } {\tilde{E}_{S} (\delta_{S} + n{\Omega \mathord{\left/ {\vphantom {\Omega 2}} \right. \kern-\nulldelimiterspace} 2})\,A_{ - n} (\varphi ,p_{H} ,p)e^{{ - in{\vartheta \mathord{\left/ {\vphantom {\vartheta 2}} \right. \kern-\nulldelimiterspace} 2}}} } ,$$

where, $\varphi = \vartheta_{H} - {\vartheta \mathord{\left/ {\vphantom {\vartheta 2}} \right. \kern-\nulldelimiterspace} 2}$ is the difference between the initial phases of the absorber vibration at the fundamental frequency and its sub-harmonic, $p_{H} = {{2\pi R_{H} } \mathord{\left/ {\vphantom {{2\pi R_{H} } {\lambda_{SC} }}} \right. \kern-\nulldelimiterspace} {\lambda_{SC} }}$ is the modulation index of the nuclear transition frequency at half of the fundamental frequency, $A_{ - n} \left( {\varphi ,p_{H} ,p} \right) = \sum\limits_{m = - \infty }^{\infty } {J_{ - n - 2m} (p_{H} )J_{m} (p)e^{ - i(n + 2m)\varphi } }$ characterizes the amplitude of the ‘*n*-th’ spectral line at frequency $\omega_{SC} + n{\Omega \mathord{\left/ {\vphantom {\Omega 2}} \right. \kern-\nulldelimiterspace} 2}$, and $\tilde{E}_{S} (\delta_{S} + n{\Omega \mathord{\left/ {\vphantom {\Omega 2}} \right. \kern-\nulldelimiterspace} 2})$ is given by (Eq. 18) in the case of Lorentz line or by Eq. (31) in the case of squared-Lorentz line. At $R_{H} = 0$ Eq. (45) turns into (Eq. 6). Correspondingly, the output spectrum of the field, the time dependence of the field, and the time dependence of the field intensity can be calculated by Eqs. (10)–(13) with substitution of Eq. (45).

**Figure 8:**
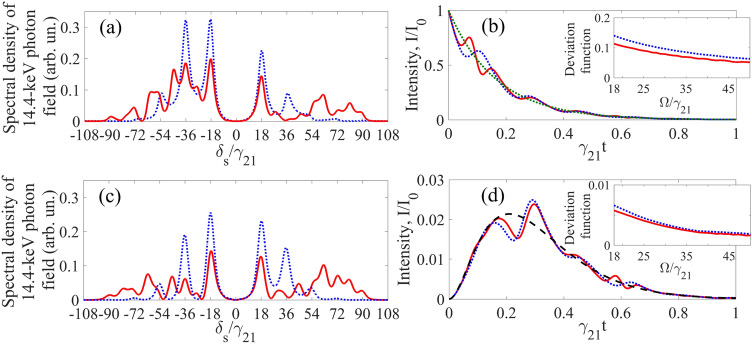
(Color online) The spectrum of the transmitted single-photon wave packet with Lorentz **(a)** and Lorentz-squared **(c)** spectral shape in the absorber reference frame for the case of single-tone vibration (blue dotted curve) and dual-tone vibration (red solid curve). The blue dotted curve in (a) is the same as the red curve in Fig. 3. The blue dotted curves in **(a)** and **(c)** are plotted as the normalized function $\left| {\tilde{E^{\prime}_{S}} (\delta_{S} ,L)} \right|^{2}$ given by Eq. (9a), where $\tilde{E}_{S} (\delta_{S} )$ is determined by Eq. (18) or Eq. (31) at $\vartheta = {\pi \mathord{\left/ {\vphantom {\pi 2}} \right. \kern-\nulldelimiterspace} 2}$, $\omega_{SC} = \omega_{21}$, $p = p_{1}$ for Lorentzian photon, **(a)**, with $\gamma_{S} = 3\gamma_{21}$, and for Lorentz-squared photon, **(c)**, with $\Delta = 0.4\gamma_{S}$ and $\gamma_{S} = 4.7\gamma_{21}$ of each line in the doublet. The vibration frequency is ${\Omega \mathord{\left/ {\vphantom {\Omega {\gamma_{21} }}} \right. \kern-\nulldelimiterspace} {\gamma_{21} }} = 18$ (corresponding to ${\Omega \mathord{\left/ {\vphantom {\Omega {\left( {2\pi } \right)}}} \right. \kern-\nulldelimiterspace} {\left( {2\pi } \right)}} \approx 10.2{\text{ MHz}}$) and $T_{M} = 5$, $T_{e} = 0$. The red solid curves in **(a)** and **(c)** are plotted as the normalized function $\left| {\tilde{E^{\prime}_{S}} (\delta_{S} ,L)} \right|^{2}$ given by Eq. (9a), where $\left| {\tilde{E^{\prime}_{S}} (\delta_{S} ,0)} \right|^{2}$ is determined by Eq. (45) at the same values as for the SMS spectral line. The fundamental frequency of the dual-tone absorber vibration is the same as that of the single-tone vibration, ${\Omega \mathord{\left/ {\vphantom {\Omega {\gamma_{21} }}} \right. \kern-\nulldelimiterspace} {\gamma_{21} }} = 18$, whereas $p_{H} = 3.1$, $p = 3.8$, $\vartheta_{H} = {\pi \mathord{\left/ {\vphantom {\pi 2}} \right. \kern-\nulldelimiterspace} 2}$, and $\varphi = - {{3\pi } \mathord{\left/ {\vphantom {{3\pi } 4}} \right. \kern-\nulldelimiterspace} 4}$. In panels **(b)** and **(d)**, there are the corresponding normalized waveforms of the Lorentz, **(b)**, and Lorentz-squared, **(d)**, photon (with deviation function (Eq. 24) in the insets) transmitted through the single-tone vibrating absorber [the blue dotted curves corresponding to the blue spectra in **(a)** and **(c)**] and dual-tone vibrating absorber [the red solid curves corresponding to the red spectra in **(a)** and **(c)**]. The curves are plotted as the function ${{I_{out} (t)} \mathord{\left/ {\vphantom {{I_{out} (t)} {I_{0} }}} \right. \kern-\nulldelimiterspace} {I_{0} }}$ [where $I_{0} = {{cE_{0}^{2} } \mathord{\left/ {\vphantom {{cE_{0}^{2} } {(8\pi )}}} \right. \kern-\nulldelimiterspace} {(8\pi )}}$] given by Eq. (12).

As discussed in previous sections, in the case of a single-tone absorber vibration at a given vibration frequency $\Omega$, the distortions of the transmitted field can be reduced (i) via reducing the total field of the wings of sidebands at the nuclear resonant frequency, $\omega_{21}$, and (ii) via reducing the effect of the nuclear quantum transition on the sidebands at the frequencies $\omega_{21} + n\Omega$. In the case of a single-tone vibration at $\gamma_{S} ,\gamma_{21} \ll \Omega$ and $p = p_{1}$, only the second option remains (i) for the Lorentz-squared photon at any initial vibration phase, $\vartheta$, of the absorber, (ii) for the Lorentz photon at $\vartheta = {\pi \mathord{\left/ {\vphantom {\pi 2}} \right. \kern-\nulldelimiterspace} 2}$, and (iii) when averaging over $\vartheta$ is realized. For a given sample of the absorber, this option can only be implemented via increasing the vibration frequency. At the same time, in the phase-locked regime of dual-tone absorber vibration, (Eq. 44), one can adjust the vibration amplitudes and phases such that $p = 3.8$, $p_{H} = 3.1$, $\vartheta_{H} = {\pi \mathord{\left/ {\vphantom {\pi 2}} \right. \kern-\nulldelimiterspace} 2}$, and $\varphi = - {{3\pi } \mathord{\left/ {\vphantom {{3\pi } 4}} \right. \kern-\nulldelimiterspace} 4}$. This results in suppression of both the resonant field and the nearest sidebands at frequencies $\omega_{21} \pm {\Omega \mathord{\left/ {\vphantom {\Omega 2}} \right. \kern-\nulldelimiterspace} 2}$ in the vibrating reference frame. Thus, the transparency spectral window is the same while the photon energy spreads further from resonance as compared to the single-tone vibration, reducing the effect of the nuclear transition on the sidebands (Fig. 8a,c). As a result, amplitude modulation in the photon waveform becomes smaller (Fig. 8b,d, with insets, red solid lines versus blue dotted lines).

As illustrated in Fig. 8a,c, adding the subharmonic to the absorber vibration noticeably alters the spectrum of the field in the vibrating reference frame and, accordingly, enriches the spectral line of the absorber with the phase-matched off-resonance sidebands in the laboratory reference frame (see Supplemental Material to^19^). At higher vibration frequencies of the absorber, this spectrum becomes similar to a spectral comb. The addition of subharmonic helps to reduce distortion in the photon waveform at relatively lower vibration frequencies when the second AIT condition in (Eq. 23) is poorly satisfied (Fig. 8b,d). As shown in the inset to Fig. 8b, for the parameter values corresponding to the boundary of the fulfillment of the AIT conditions (Eq. 23), the deviation function (Eq. 24) for the Lorentz photon decreases by 22% from $D \approx 0.141$ for single-tone absorber vibration to $D \approx 0.11$ for dual-tone vibration. For the Lorentz-squared photon in the inset to Fig. 8d, the deviation function is reduced by 13% from $D \approx 6.65 \times 10^{ - 3}$ for single-tone absorber vibration to $D \approx 5.8 \times 10^{ - 3}$ for dual-tone vibration. At relatively high vibration frequencies, when conditions (Eq. 23) are well met, the single-tone vibration alone is sufficient to ensure high transparency.

In the random-phase regime of the dual-tone absorber vibration, the regular amplitude modulation of the photon waveform disappears for both Lorentz and Lorentz-squared photons similar to the case of the single-tone vibration. The addition of the subharmonic reduces the deviation function by about 14% for Lorentz photon and by 9% for Lorentz-squared photon at the same parameter values as in Fig. 8.

## Conclusion

In this paper, we have extended the phenomenon of acoustically induced transparency (AIT) into the novel phase-locked regime, when the transmitted photons are synchronized with the absorber vibration. This regime is the most promising for realization of the acoustically controlled quantum interfaces between individual x-ray photons and nuclear ensembles.

We have shown that the synchrotron Mössbauer sources (SMS) provide an excellent opportunity for realization of this regime due to periodic nature of the radiation pulses and their deterministic timing, variable shape and duration of the single-photon wave packet, high photon fluxes and tight focusing the photon beam.

We have found that in the phase-locked regime of AIT, the transmitted photon waveform (the time dependence of the photon detection probability, proportional to the photon count rate or the intensity of the single-photon wave packet) acquires regular amplitude modulation, inherent to this regime due to interference effects during the photon propagation. The corresponding changes appear also in the transmitted photon spectrum. At the fixed absorber vibration frequency, the maximum amplitude of the photon waveform modulation depends on the initial phase of the absorber vibration and can be minimized by the proper choice of this phase. The waveform modulation amplitude can be reduced by increasing the vibration frequency. In the case of a relatively low absorber vibration frequency, amplitude modulation of the transmitted photon waveform and spectrum distortions can be reduced by using dual-tone vibration. We have shown also that both shorter single-photon pulse duration of SMS as compared to radioactive Mössbauer source and Lorentz-squared spectral profile of the single-photon wave packet available with SMS are favorable for better preservation of the spectral-temporal characteristics of the photon.

The main conclusion is that high-quality AIT in the phase-locked regime may be implemented with SMS at the ESRF and Spring-8 facilities in the readily available range of parameters of these sources.
